# Contemporary menopausal hormone therapy and risk of cardiovascular disease: Swedish nationwide register based emulated target trial

**DOI:** 10.1136/bmj-2023-078784

**Published:** 2024-11-27

**Authors:** Therese Johansson, Torgny Karlsson, Dana Bliuc, Daniel Schmitz, Weronica E Ek, Alkistis Skalkidou, Jacqueline R Center, Åsa Johansson

**Affiliations:** 1 Department of Immunology, Genetics and Pathology, SciLifeLab, Uppsala University, Uppsala, Sweden; 2 Centre for Women’s Mental Health during the Reproductive Lifespan – Womher, Uppsala University, Uppsala, Sweden; 3 Garvan Institute of Medical Research, Sydney, NSW, Australia; 4 Clinical School, St Vincent's Hospital, Faculty of Medicine and Health, University of New South Wales Sydney, Sydney, NSW, Australia; 5 Department of Women’s and Children’s Health, Uppsala University, Uppsala, Sweden

## Abstract

**Objective:**

To assess the effect of contemporary menopausal hormone therapy on the risk of cardiovascular disease according to the route of administration and combination of hormones.

**Design:**

Nationwide register based emulated target trial.

**Setting:**

Swedish national registries.

**Participants:**

919 614 women aged 50-58 between 2007 and 2020 without hormone therapy use in the previous two years, identified from the Swedish population.

**Interventions:**

138 nested trials were designed, starting each month from July 2007 until December 2018. Using the prescription registry data for that specific month, women who had not used hormone therapy in the previous two years were assigned to one of eight treatment groups: oral combined continuous, oral combined sequential, oral unopposed oestrogen, oral oestrogen with local progestin, tibolone, transdermal combined, transdermal unopposed oestrogen, or non-initiators of menopausal hormone therapy.

**Main outcome measures:**

Hazard ratios with 95% confidence intervals were estimated for venous thromboembolism, as well as for ischaemic heart disease, cerebral infarction, and myocardial infarction separately and as a composite cardiovascular disease outcome. Treatment effects were estimated by contrasting initiators and non-initiators in observational analogues to “intention-to-treat” analyses and continuous users versus never users in “per protocol” analyses.

**Results:**

A total of 77 512 women were initiators of any menopausal hormone therapy and 842 102 women were non-initiators. 24 089 women had an event recorded during the follow-up: 10 360 (43.0%) had an ischaemic heart disease event, 4098 (17.0%) had a cerebral infarction event, 4312 (17.9%) had a myocardial infarction event, and 9196 (38.2%) had a venous thromboembolic event. In intention-to-treat analyses, tibolone was associated with an increased risk of cardiovascular disease (hazard ratio 1.52, 95% confidence interval 1.11 to 2.08) compared with non-initiators. Initiators of tibolone or oral oestrogen-progestin therapy had a higher risk of ischaemic heart disease (1.46 (1.00 to 2.14) and 1.21 (1.00 to 1.46), respectively). A higher risk of venous thromboembolism was observed for oral continuous oestrogen-progestin therapy (1.61, 1.35 to 1.92), sequential therapy (2.00, 1.61 to 2.49), and oestrogen-only therapy (1.57, 1.02 to 2.44). Additional results in per protocol analyses showed that use of tibolone was associated with a higher risk of cerebral infarction (1.97, 1.02 to 3.78) and myocardial infarction (1.94, 1.01 to 3.73).

**Conclusions:**

Use of oral oestrogen-progestin therapy was associated with an increased risk of heart disease and venous thromboembolism, whereas the use of tibolone was associated with an increased risk of ischaemic heart disease, cerebral infarction, and myocardial infarction but not venous thromboembolism. These findings highlight the diverse effects of different hormone combinations and administration methods on the risk of cardiovascular disease.

## Introduction

Cardiovascular disease is the leading cause of death globally, contributing to approximately 30% of all deaths.^1^ Women tend to develop cardiovascular disease several years later than men do, with a notable increase during midlife, a period coincident with the menopausal transition.^2^ This transition is characterised by a decline in oestrogen and an increase in follicle stimulating hormone concentrations. These hormonal changes can induce effects on the neuroendocrine system, resulting in hot flushes, night sweats, sleep disorders, anxiety, and memory loss. As the world population ages, a significant demographic shift places a larger fraction of women in the postmenopausal phase. Managing this phase, marked by decreased oestrogen concentrations and various menopausal symptoms, largely relies on systemic menopausal hormone therapy. Systemic menopausal hormone therapy is based on administration of oestrogen with or without the addition of progestogens, effectively mitigates vasomotor symptoms,^3^ and reduces the incidence of vertebral and hip fractures.^4^ ^5^ However, although menopausal hormone therapy alleviates menopausal symptoms, studies have suggested an association between its use and an increased risk of cardiovascular disease.^6^ ^7^ ^8^ ^9^ ^10^

Experimental studies have shown that oestrogen plays a protective role in cardiovascular health by promoting angiogenesis and vasodilation,^11^ ^12^ reducing cardiac fibrosis and oxidative stress and increasing high density lipoprotein cholesterol concentrations. 13 14 15 More than two decades ago, observational studies conducted through the Nurses’ Health Study in the US and the General Practice Research Database in the UK supported a potential cardiovascular benefit for postmenopausal women using hormone therapy.^16^ ^17^ ^18^ However, subsequent randomised trials, including the Heart and Oestrogen/Progestin Replacement Study (HERS) and the Women’s Health Initiative trial, showed the opposite.^19^ ^20^ ^21^ ^22^ HERS showed no reduction in coronary heart disease rates and an increase in coronary events and myocardial infarctions in the treatment group. Similarly, the Women’s Health Initiative found increased risks of coronary heart disease, stroke, and pulmonary embolism, leading to the conclusion that the risks of hormone therapy outweighed the benefits, especially because of the increased risk of stroke. These trials challenged the initial positive outlook inspired by observational studies and altered our comprehension of the complexities and associated risks of menopausal hormone therapy, prompting a critical reassessment of its usage.

The discrepancy between previous observational studies and randomised controlled trials has been attributed to population differences and methodological biases.^23^ ^24^ In most observational studies, hormone use started around menopause, whereas 67% of Women’s Health Initiative participants were aged over 60. Observational studies compared prevalent users with non-users, introducing a selection bias as tolerant women are included in the user group with depletion of women susceptible to cardiovascular disease.^25^ Moreover, observational estimates are heavily weighted by long term use and would have missed any increased risk seen at the beginning of treatment even if only new users had been analysed. Analysing observational data by using a design that emulates a target trial has been suggested to mitigate such pitfalls.^26^ Other than the randomisation, which is not possible in an observational study, emulating a target trial is advocated as being more accurate for causal inference than a traditional observational study.^27^ In a randomised trial, time zero aligns the start of follow-up, treatment assignment, and evaluation of eligibility simultaneously. Emulating this framework by using observational data helps to avoid common biases, including immortal time bias and selection bias, by ensuring that all critical processes are aligned at the same starting point (time zero). By applying this method, data from the Nurses’ Health Study were reanalysed to resemble the Women’s Health Initiative trial.^16^ ^17^ Unlike the original analyses, the revised effect estimates aligned with the Women’s Health Initiative, probably owing to reduced selection bias.^28^

Two decades have passed since the publication of the HERS and the Women’s Health Initiative trials. Since then, new menopausal hormone therapy products with different formulations and routes of administration have been introduced to the market. Both the HERS and the Women’s Health Initiative investigated the risk of cardiovascular disease associated with only one type of menopausal hormone therapy, specifically oral conjugated equine oestrogen combined with medroxyprogesterone acetate. In addition, HERS and the Women’s Health Initiative included women with an average age of 67 and 63, respectively, which is not the typical timing for starting menopausal hormone therapy today. A critical need exists for further studies to investigate the effects of contemporary menopausal hormone therapy in a clinically relevant population.^29^

To tackle the gaps in the knowledge on use of contemporary menopausal hormone therapy and risk of cardiovascular disease, this paper reports the findings from a Swedish nationwide register based emulated target trial including 919 614 women aged 50-58 years. This age range represents the typical span during which women undergo the menopausal transition—a period marked by significant hormonal changes and when most women start menopausal hormone therapy. We explored a variety of menopausal hormone therapy formulations, including oral and transdermal options, combined therapies with progestin, oestrogen-only treatments, and tibolone. These diverse options mirror the range of choices available in clinical practice, tailored to meet the needs and preferences of individual patients. Different hormone administration methods are thought to have distinct physiological effects. Oral oestrogens increase production of coagulation factor, suggested to have a less favourable cardiovascular risk profile,^30^ whereas transdermal administration bypasses the liver and is believed to be a better option.^31^ The addition of progestins in combined preparations may increase the oestrogenicity and, therefore, the risk of thrombosis.^32^

By clearly defining the target trial protocol and its observational emulation, we ensured eligibility and treatment alignment from the start, preventing selection bias by excluding prevalent users at baseline.^33^ We aimed to estimate the average treatment effect for ischaemic heart disease, cerebral infarction, myocardial infarction, and venous thromboembolism with the use of different types of contemporary menopausal hormone therapy.

## Methods

### Study population

We identified the population on the basis of data provided by Statistics Sweden.^34^ A unique personal identification number that is given to all Swedish citizens at birth and to people who have immigrated to Sweden is used in all public registries, allowing reliable linkage of data among different registries. Statistics Sweden also provided data on the highest achieved level of education, emigration, and ancestral origin. The Swedish National Board of Health and Welfare provided data from the Swedish prescribed drug register, the national patient register, the cancer register, and the cause of death register.^35^

We designed this observational analysis to emulate a target trial (that is, a hypothetical pragmatic trial that would have answered the causal question of interest) of the effect of menopausal hormone therapy compared with no menopausal hormone therapy on the risk of cardiovascular disease outcomes. Table 1 shows the protocol of the target trial and its observational emulation.

**Table 1:** Protocol for emulation of target trial

Protocol components	Target trial	Emulation using observational data
Eligibility criteria	Postmenopausal women aged 50-58 years	Similar to target trial
	No use of menopausal hormone therapy within previous 2 years	Data lookback* 2 years
	No previous history of cardiovascular disease	Data lookback ≥20 years
	No previous history of cancer	Data lookback ≥49 years
	No previous history of hysterectomy or bilateral oophorectomy surgery	Data lookback ≥11 years
Treatment strategy	Start menopausal hormone therapy at baseline and continue until end of follow-up. Refrain from starting menopausal hormone therapy during follow-up	Same as target trial
Assignment procedure	Study participants will be randomly assigned to either strategy at baseline	Participants are assigned on basis of their observed data at baseline. Randomisation is emulated by inverse probability weighting
Follow-up period	Starts at randomisation and ends at diagnosis of cardiovascular disease, death, emigration, or 2 years after baseline, whichever occurs first	Same as target trial
Outcome	First time diagnosis of composite cardiovascular disease and cause specific cardiovascular disease	Similar to target trial
Causal contrast of interest	Intention-to-treat effect; per protocol effect	Same as target trial
Analysis plan	Intention-to-treat effect estimated via comparison of 2 year cardiovascular disease risk among women assigned to each treatment strategy. Per protocol effect estimated via comparison of 2 year cardiovascular disease risk among women assigned to and continuously following assigned treatment strategy. Per protocol effects adjusted for pre-baseline and post-baseline confounders associated with adherence to strategies of interest. All analyses adjusted for all known confounders	Intention-to-treat effect and per protocol effect. All analyses adjusted for measured baseline potential confounders by inverse probability weighting and for censoring due to loss to follow-up. Per protocol effect adjusted for pre-baseline and post-baseline measured confounders associated with adherence to strategies of interest by inverse probability weighting
Analysis	Hazard ratios and Kaplan-Meier survival curves estimated from Cox proportional hazard models	Hazard ratios and adjusted survival curves estimated via Cox proportional hazard models

* Refers to period for which historical data are available from start of our study. For instance, first trial began in 2005; with 20 year lookback in patient register, data were accessed from 1985.

### Eligibility criteria and washout period

We designed 138 nested trials, with one trial starting each month during the study period—that is, from July 2007 until December 2018. Here, the month of start of follow-up is termed the “trial month” and equals July 2007 for the first trial, August 2007 for the second trial, and so on. In each trial, we identified all women who, before the start of follow-up, were between 50 and 58 years of age and had not redeemed a prescription for any menopausal hormone therapy in the previous two years (that is, the washout period). We excluded women who, before the start of follow-up, had a previous history of ischaemic heart disease, stroke, peripheral vascular disease, peripheral arterial disease, or cancer. We also excluded women who had undergone bilateral oophorectomy, unilateral oophorectomy twice, hysterectomy, or a sterilisation procedure. Our exclusion criteria included diagnoses recorded in the patient register from 1987, causes of death from 1952, and cancer diagnoses from 1958, ensuring comprehensive coverage of almost all significant medical events throughout the lifetime of participants included in the study. The relevant diagnostic, surgical, and medication codes used for exclusion are listed in supplementary table S1. We excluded women who appeared for the first time or with a new personal identification number in the registers after 2005. This group represents either people who immigrated to Sweden or women who had received gender affirming care. This was due to missing pre-immigration medical data and potential confounding factors from gender transition procedures.

### Sequence of pragmatic trials

**Fig 1:**
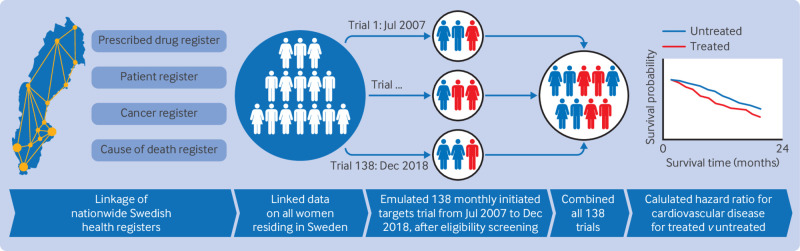
Schematic illustration of emulated target trial design. Nationwide Swedish registers, including prescribed drug register, patient register, cancer register, and cause of death register were linked. After eligibility screening, 138 monthly initiated target trials from 2007 to December 2018 were emulated. All 138 trials were combined to calculate hazard ratios for cardiovascular disease and venous thromboembolism comparing women starting menopausal hormone therapy versus untreated women. Survival curve shown in figure is for illustrative purposes only and does not represent actual study results

The small number of hormone therapy initiators (n=331) and cardiovascular disease events (n=8) in the first trial makes conducting a meaningful analysis from one single trial impossible. Instead, we emulated 138 trials, one trial each month during the study period, with each trial having a one month enrolment period. This ensures that all eligible initiators and events are included in the analysis. This approach increases statistical power compared with selecting only one of those instances as time zero and accounts for the fact that individuals can meet eligibility criteria multiple times during the study period^36^; figure 1 illustrates the study design. The emulation of a series of trials, such that each individual may participate in multiple trials, has been successfully applied in previous studies when comparing treatment versus non-treatment.^37^ ^38^ ^39^ ^40^ ^41^ Supplementary table S2 shows the numbers of participants, initiators, events by “trial,” and people who did not meet eligibility criteria (see previous paragraph).

### Study outcomes

We obtained the date of disease diagnoses from the national patient register and the cause of death register, which collects data on discharge diagnoses from public and private Swedish hospitals. The specific outcomes of this study (supplementary table S3) included ischaemic heart disease (ICD-10 (international classification of diseases, 10th revision) codes: I20, I21, I22, I24, I25), myocardial infarction (ICD-10 codes: I21, I22), cerebral infarction (ICD-10 code: I63), and venous thromboembolism (ICD-10 codes: I26, I80, I81, I82). We analysed these diseases as separate outcomes and as a composite outcome defined as the first occurrence of any cardiovascular disease (ICD-10 codes: I20, I21, I22, I24, I25, I63).

### Treatment groups and follow-up

We retrieved the redeemed prescriptions for the study period from the prescribed drug register, which categorises information according to Anatomical Therapeutical Chemical codes. The relevant Anatomical Therapeutical Chemical codes for systemic menopausal hormone therapy products are listed in supplementary table S4. Additional information on name, trade name, dose, number of packages, defined daily doses per package, tablets per package, route of administration, and date of redemption was available. We classified eligible women into one of eight treatment strategies according to their redeemed or non-redeemed prescriptions at baseline: initiators who started oral combined continuous therapy, oral combined sequential therapy, oral unopposed oestrogen, oral oestrogen combined with the levonorgestrel intrauterine system, tibolone, transdermal combined therapy, or transdermal unopposed oestrogen and non-initiators who did not start any menopausal hormone therapy during the “trial month.” We considered contemporary redemption of unopposed oestrogen and progestogen (natural or synthetic) as one of two “self-combined therapies,” depending on the ratio between the oestrogen and progestogen dose: continuous combined therapy if the ratio was <7 and sequential combined therapy if the ratio was >7. We further subdivided the categorisation of “self-combined” according to the route of administration (oral or dermal). If a woman combined transdermal oestrogen with an oral, transdermal, or local progestogen, we categorised this woman as transdermal combined. We assumed that women who inserted a levonorgestrel intrauterine system during the trial month had it for the entire follow-up.

The total follow-up period for each trial was set to two years. We followed individuals until the first occurrence of the study endpoint, death, emigration, or end of follow-up (that is, two years), whichever occurred first.

### Covariates

We achieved adjustment for potential confounding, measured at the baseline of each trial, by using inverse probability of treatment weighting. We used the directed acyclic graph approach to identify potential confounders and unmeasured confounders (supplementary figure S1). We added age at baseline to account for differences in cardiovascular disease risk. We included trial month as a continuous term (1-138) to account for potential differences in offset between trials, thereby increasing precision in effect estimates. We included calendar year to adjust for trends in menopausal hormone therapy prescription patterns and changes in cardiovascular disease prevalence during the study period. We used the level of education as a proxy for socioeconomic status. We adjusted for geographical regions to account for differences in prescription patterns between rural and urban regions. We included region of birth to account for potential differences in exposure and outcome. To account for differences in predisposing diseases and disorders between initiators and non-initiators, we adjusted for hypertension, heart disease, and diabetes (defined by the use or non-use of drugs for these conditions). For covariate adjustment, inverse probability of treatment weighting, relevant Anatomical Therapeutical Chemical codes, and data variables, see supplementary methods and table S5.

### Causal contrasts of interest

We used observational analogues of intention-to-treat and per protocol analyses to estimate the effect of menopausal hormone therapy on risk of cardiovascular disease and venous thromboembolism. In the intention-to-treat model, we compared the incidence rate of cardiovascular disease in the initiator groups versus the non-initiator group. In the intention-to-treat analyses, we did not consider whether women who started treatment at baseline did not redeem any new prescriptions or altered their prescriptions or whether non-initiators started any menopausal hormone therapy during the follow-up. In the per protocol analyses, we censored initiators when they deviated from the protocol; that is, if they discontinued treatment or changed preparation. Similarly, we censored non-initiators when they deviated from the protocol; that is, if they started any treatment during the follow-up. Thus, in the per protocol analyses, all initiators were continuous users and all non-initiators were never users.

### Statistical analysis

We used cause specific Cox proportional hazard modelling to estimate the hazard ratio of the composite cardiovascular disease outcome, with “time since the start of follow-up” as the timescale and a time fixed binary variable for menopausal hormone therapy initiation (based on information from the prescribed drug register). We also estimated the effect of initiation of menopausal hormone therapy on the hazard rate of the specific outcomes: ischaemic heart disease, cerebral infarction, myocardial infarction, and venous thromboembolism. The cause specific hazard model allows us to investigate the rate of occurrence of specific events, such as myocardial infarction, while treating other potential outcomes as competing risks. In this framework, events such as other forms of ischaemic heart disease (excluding myocardial infarction), cerebral infarction, and venous thromboembolism are considered competing risks. When these events occur, participants are censored at that time, meaning that they are removed from the analysis for the primary event of interest, myocardial infarction. However, when analysing ischaemic heart disease and myocardial infarction separately, we deliberately do not censor the events coded as myocardial infarction under ICD-10 codes I21 and I22.^42^ Estimated hazard ratios in the main results are average contrasts over the follow-up of two years.

Additionally, we calculated the adjusted incidence rates for different follow-up periods, as shown in supplementary table S6. We did sensitivity analyses using Fine and Gray subdistribution models to account for competing risks between specific cardiovascular diseases, death, and emigration.^43^ We achieved this by including in the risk set individuals who died or experienced another competing event, without censoring them. We note that a cause specific hazard ratio may be interpreted as a direct effect of the treatment on the outcome, whereas a subdistribution hazard ratio denotes the total effect of the treatment.^44^ We fitted Cox regression models by using the coxph function and Fine and Gray models by using the finegray function, both available in the survival package (v3.5-7) in R. We used the common α level of 0.05 as a threshold for significance.

We used stabilised weights to adjust for confounding in the intention-to-treat and per protocol analyses. As a result, marginal effects are estimated corresponding to the average treatment effect. We included only baseline covariates in the intention-to-treat analyses, whereas we also included time varying covariates in the per protocol analyses. In the per protocol analyses, we estimated inverse probability weights to adjust for potential selection bias introduced by artificial censoring.^45^ See supplementary methods for details.

We plotted adjusted cumulative incidence curves for the intention-to-treat analysis by using inverse probability of treatment weighting for covariate adjustment. We used the adjustedCurves R package to visualise the probability of the event of interest over the two year follow-up period.^38^ To quantify the effect of different treatments on the event of interest, we calculated the absolute risk difference after one year of follow-up.^38^ This analysis was limited to menopausal hormone therapies that showed a significant difference compared with the non-initiator group.

In the intention-to-treat analyses, we compared the incidence rate of cardiovascular disease in the initiator groups versus the non-initiator group. In the per protocol analyses, we extended all prescription periods to twice that of the previous redeemed package, as has been suggested in previous studies.^46^ For example, if a package included 42 tablets with one tablet taken each day according to the defined daily doses, the exposure time was extended by 84 days from the last day of the preceding prescription. We calculated the month of last menopausal hormone therapy use by using the “compute.treatment.episode” function in the AdhereR package (v0.8.1) in R.^47^

Because the sequence of 138 trials started every month, many women participated in more than one trial. To adjust for their events being recorded more than once, we used the robust variance estimator to estimate the conservative 95% confidence intervals (CIs) by adding a cluster(id) term to the models.^48^ Women who had inserted a levonorgestrel intrauterine system before 2005 would have been categorised as non-initiators or, if they were using menopausal hormone therapy, as initiators of “unopposed oestrogen” (transdermal or oral). Consequently, we did a sensitivity analysis with a six year washout period to prevent potential exposure misclassification. We used R (version 4.2.2) for all analyses.

### Patient and public involvement

Our study considers an important public health question. As the study is register based, no direct contact with the patients or participants included in the research occurred at any stage. However, interactions with patients and via public media indicate that the lack of knowledge about the risks associated with different types of menopausal hormone therapies leaves women of perimenopausal and postmenopausal age feeling very uncertain about their medication choices. This lack of awareness served as the primary motivation for conducting the study. Additionally, our team includes a gynaecologist who actively works with patients, providing firsthand insights into the clinical aspects of menopause treatment. Several of the co-authors have either personal or close family experiences with menopause, enhancing our study’s depth and relevance through lived experiences. Unfortunately, the lack of funding specifically allocated for patient and public involvement prevented us from engaging patients or the public in setting the research questions, designing the study, or interpreting and writing up the results. To increase awareness of these results to the public, the results will be presented as a press release and shared across social media. Results will also be presented to students and incorporated into the national clinical guidelines for menopausal hormone therapy use.

## Results

**Fig 2:**
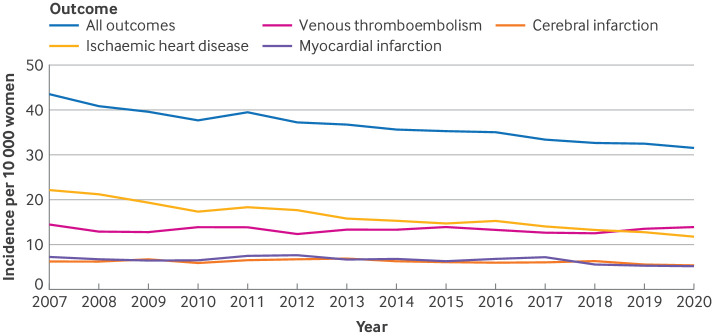
Trends in incidence of cardiovascular disease in Sweden among women aged 50-58 years. Incidence of ischaemic heart disease, cerebral infarction, myocardial infarction, venous thromboembolism, and all outcomes together. Data from cause of death register and patient register

The eligibility criteria for at least one of the 138 trials were met by 919 614 women, of whom 24 089 had an event recorded during the follow up (two years from baseline). For the disease specific events, 10 360 (43.0%) women had an ischaemic heart disease event, 4098 (17.0%) had a cerebral infarction event, 4312 (17.9%) had a myocardial infarction event, and 9196 (38.2%) had a venous thromboembolic event. Between 2007 and 2020, a significant reduction of more than 50% in the incidence of cardiovascular disease occurred among women aged 50-58 years. The most substantial decline was for ischaemic heart disease, which dropped from 22.2 per 10 000 women in 2007 to 11.8 in 2020. The incidence of venous thromboembolism remained stable during the study period, with an incidence of 14.5 per 10 000 women in 2007 and 13.9 in 2020 (fig 2).

**Fig 3:**
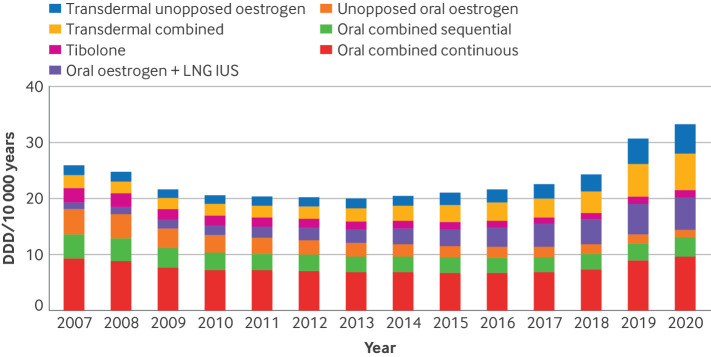
Trends in menopausal hormone therapy use in Sweden among women aged 50-58 years. Defined daily dosages (DDD) of different types of menopausal hormone therapies from 2007 to 2020. LNG IUS=levonorgestrel intrauterine system

A total of 77 512 women were initiators of any menopausal hormone therapy and 842 102 women were non-initiators. The most frequently initiated form of menopausal hormone therapy was oral combined continuous therapy, making up more than a third of all initiated treatments. Transdermal therapies were the next most common, accounting for more than a fifth of all initiated treatments. During the study period, we saw a 50% increase in the use of transdermal menopausal hormone therapy products and more than a 50% decrease in unopposed oral oestrogen and tibolone (fig 3).

Compared with non-initiators, women who initiated menopausal hormone therapy were slightly younger (mean age 53.0 *v* 53.9), had a higher level of education, resided more frequently in urban regions (39.8% *v* 32.3%), and redeemed more prescriptions for heart disease medications (13.5% *v* 10.7%) and hypertension medications (29.7% *v* 27.0%), but a smaller proportion had prescriptions for diabetic medication (5.8% *v* 7.8%). Among those who started menopausal hormone therapy, initiators of oral oestrogen plus levonorgestrel intrauterine system had the lowest mean age (51.8), whereas those using tibolone were the oldest (mean age 53.7). Women who initiated unopposed oral oestrogen tended to have lower education and were more likely to reside in rural regions (table 2). We adjusted for these differences in our analyses.

**Table 2:** Baseline characteristics of initiators and non-initiators and of different menopausal hormone therapy groups. Values are numbers (percentages) unless stated otherwise

Characteristics	Initiators	Non-initiators	Oral combined continuous	Oral combined sequential	Unopposed oral oestrogen	Oral oestrogen+LNG IUS	Tibolone	Transdermal combined*	Transdermal unopposed oestrogen
Mean (SD) age, years	53.0 (2.3)	53.9 (2.6)	53.4 (2.3)	52.2 (1.9)	53.4 (2.5)	51.8 (1.8)	53.7 (2.4)	52.9 (2.3)	52.9 (2.4)
**Highest educational level**									
Elementary school	7178 (9.26)	107 789 (12.8)	2998 (10.5)	1489 (9.46)	573 (12.8)	228 (5.30)	535 (8.85)	921 (7.54)	434 (7.04)
High school	35 332 (45.6)	399 073 (47.4)	13 757 (48.1)	7115 (45.2)	2160 (48.3)	1886 (43.9)	2582 (42.7)	5214 (42.7)	2618 (42.4)
High school and higher education	33 840 (43.7)	322 525 (38.3)	11 47 1(40.1)	6889 (43.8)	1654 (37.0)	2125 (49.4)	2824 (46.7)	5856 (48.0)	3021 (49.0)
Research education	983 (1.27)	8926 (1.06)	287 (1.00)	208 (1.32)	53 (1.19)	60 (1.40)	87 (1.44)	200 (1.64)	88 (1.43)
Unknown	179 (0.23)	3789 (0.45)	65 (0.23)	38 (0.24)	31 (0.69)	<5 (0.02)	16 (0.26)	20 (0.16)	8 (0.13)
**Region of birth**									
Nordics	68 396 (88.2)	731 281 (86.8)	25 341(88.7)	13517 (85.9)	3928 (87.9)	3978 (92.5)	5250 (86.9)	10789(88.4)	5593 (90.7)
Africa	427 (0.55)	7663 (0.91)	147 (0.51)	103 (0.65)	26 (0.58)	13 (0.30)	34 (0.56)	67 (0.55)	37 (0.60)
Asia	2797 (3.61)	37 305 (4.43)	972 (3.40)	813 (5.17)	169 (3.78)	80 (1.86)	235 (3.89)	390 (3.19)	138 (2.24)
Europe without Nordics	4643 (5.99)	55 326 (6.57)	1699 (5.95)	1025 (6.51)	256 (5.73)	189 (4.40)	388 (6.42)	782 (6.40)	304 (4.93)
North America	321 (0.41)	2863 (0.34)	93 (0.33)	72 (0.46)	33 (0.74)	14 (0.33)	23 (0.38)	60 (0.49)	26 (0.42)
Oceania	27 (0.03)	252 (0.03)	12 (0.04)	5 (0.03)	<5 (0.04)	<5 (0.02)	<5 (0.05)	<5 (0.02)	<5 (0.03)
South America	901 (1.16)	7410 (0.88)	314 (1.10)	204 (1.30)	57 (1.27)	25 (0.58)	111 (1.84)	121 (0.99)	69 (1.12)
**Medications**									
Diabetes	4518 (5.83)	65 599 (7.79)	1654 (5.79)	906 (5.76)	400 (8.95)	190 (4.44)	338 (5.6)	683 (5.6)	333 (5.4)
Heart disease	10 464 (13.5)	90 104 (10.7)	3858 (13.5)	2140 (13.6)	563 (12.6)	516 (12.0)	864 (14.3)	1709 (14.0)	838 (13.6)
Hypertension	23 021 (29.7)	227 367 (27.0)	8430 (29.5)	4658 (29.6)	1560 (34.9)	1272 (29.6)	1861 (30.8)	3528 (28.9)	1733 (28.1)
None	39 509 (50.9)	460 542 (54.6)	14 636 (51.2)	8035 (51.1)	1948 (43.6)	2322 (54.0)	2981 (49.4)	6291 (51.4)	3265 (53.0)
**Geographical region**									
Urban	30 849 (39.8)	271 998 (32.3)	9859 (34.5)	6106 (38.8)	1372 (30.7)	1646 (38.3)	3185 (52.7)	6081 (49.8)	3022 (49.0)
Semi-urban	6433 (8.3)	88 420 (10.5)	2943 (10.3)	1333 (8.47)	464 (10.4)	285 (6.65)	244 (4.05)	660 (5.41)	463 (7.52)
Semi-rural	29 764 (38.4)	348 630 (41.4)	11 316 (39.6)	6028 (38.3)	1850 (41.4)	1681 (39.1)	2242 (37.1)	4090 (33.5)	2122 (34.4)
Rural	10 541 (13.6)	133 894 (15.9)	4460 (15.6)	2272 (14.4)	785 (17.4)	688 (15.9)	373 (5.51)	1380 (11.2)	562 (9.09)

Exact numbers have not been reported for categories with fewer than five individuals. These counts are represented as <5 to maintain data confidentiality.

LNG=levonorgestrel; IUS=intrauterine system.

* Includes women who combined transdermal oestrogen with an oral, transdermal, or local progestogen.

### Intention-to-treat estimates of effect of menopausal hormone therapy on cardiovascular disease

When we pooled the participants across all “trials,” we had 50 732 837 person trials and a total of 385 952 events were recorded during the follow-up. During 154 433 person years, we observed 664 events among initiators of menopausal hormone therapy, with an adjusted incidence rate of 4.37per 1000 person years. The corresponding incidence rate in 101 million person years contributed by non-initiators, during which 385 288 women had a cardiovascular event, was 3.56 per 1000 person years. The higher number of person years in non-initiators is mainly owing to many of them meeting eligibility criteria multiple times and being included in multiple trials. By contrast, initiators will be ineligible from at least the subsequent 25 trials because they had a redeemed prescription for menopausal hormone therapy during the two year washout period.

**Fig 4:**
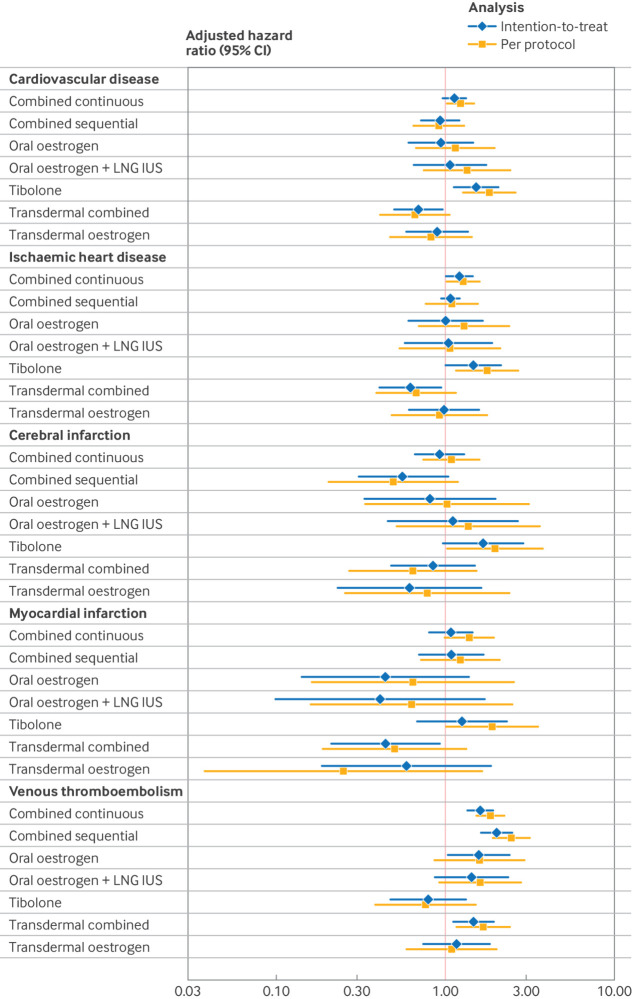
Forest plot for hazard ratios of cardiovascular disease in two causal contrasts; intention-to-treat (blue) and per protocol (orange) analysis. Intention-to-treat analyses were adjusted for baseline covariates (age, calendar year, trial month, education level, residence of living, region of birth, and medication use for heart disease, diabetes, and hypertension) by stabilised inverse probability for treatment weights. Per protocol analyses were adjusted for same baseline confounders as intention-to-treat analyses and for time varying confounding by stabilised inverse probability of treatment weights and inverse probability of censoring weights to adjust for artificial censoring of individuals when they deviated from protocol. Estimated hazard ratios are average contrasts over follow-up of two years. CI=confidence interval; LNG IUS=levonorgestrel intrauterine system

The estimated hazard ratio of cardiovascular disease among initiators of oral combined therapy, unopposed oral oestrogen, or oral oestrogen plus levonorgestrel intrauterine system did not differ significantly compared with non-initiators, with estimates ranging from 0.93 to 1.13. We observed no increased risk in women using transdermal combined or transdermal unopposed oestrogen menopausal hormone therapy. Initiating tibolone was associated with an increased risk of the composite cardiovascular disease outcome (hazard ratio 1.52, 95% CI 1.11 to 2.08) (fig 4; supplementary table S7). The calculated cumulative risk difference between tibolone initiators and non-initiators over one year was 0.001 (supplementary figure S2A). This means that for every 1000 women who start taking tibolone during a year, one will develop a cardiovascular disease such as ischaemic heart disease, myocardial infarction, or cerebral infarction.

For the individual diseases, initiating oral combined continuous therapy or tibolone was associated with higher risk of ischaemic heart disease, with hazard ratios of 1.21 (95% CI 1.00 to 1.46) and 1.46 (1.00 to 2.14), respectively. The cumulative risk difference compared with non-initiators was 0.0099 and 0.0012, respectively (supplementary figure S2B). This translates to approximately 11 new cases of ischaemic heart disease per 1000 women who start treatment with oral combined continuous therapy or tibolone over one year. Furthermore, we observed an increased risk of venous thromboembolism with several hormone therapies: oral combined continuous (hazard ratio 1.61, 95% CI 1.35 to 1.92), oral combined sequential (2.00, 1.61 to 2.49), unopposed oral oestrogen (1.57, 1.02 to 2.44), and transdermal combined (1.46, 1.09 to 1.95). The cumulative risk differences were 0.0017 for oral combined continuous, 0.0025 for oral combined sequential, 0.0018 for unopposed oral oestrogen, and 0.001 for transdermal combined therapy (supplementary figure S2E). If 1000 women started each of these treatments and were observed for a year, we would expect to see seven new cases of venous thromboembolism across all groups.

Our estimates remained similar when we used the Fine and Gray subdistribution hazard model (supplementary table S8). In our sensitivity analysis, which included a six year washout period, our estimates remained largely unchanged (see supplementary table S9). However, because this approach excluded a significant portion of data, it resulted in wider confidence intervals. Additionally, for some outcomes, the number of events was too low—or in some cases, non-existent—to allow for a meaningful analysis.

### Per protocol estimates of effect of menopausal hormone therapy on cardiovascular disease

The estimated intention-to-treat effect is influenced by the participant’s adherence to the treatment strategy allocated at the start of the trial. Thus, intention-to-treat comparisons might underestimate the effect that would have been observed if all participants had fully adhered to their assigned treatments.^49^ In our study, 46.4% of initiators discontinued their assigned treatment within one year and 63.7% discontinued within two years, and 3.1% of non-initiators started taking some systemic menopausal hormone therapy within two years. For this reason, we also did per protocol analyses. We saw 378 478 incident events in never users and 444 in users during 99.5 million person years of observation.

We observed an increase in the risk associated with oral combined continuous therapy (hazard ratio 1.22, 95% CI 1.00 to 1.50). Consistent with our intention-to-treat analysis, women using tibolone had a higher hazard rate of cardiovascular disease (1.81, 1.25 to 2.61).

**Fig 5:**
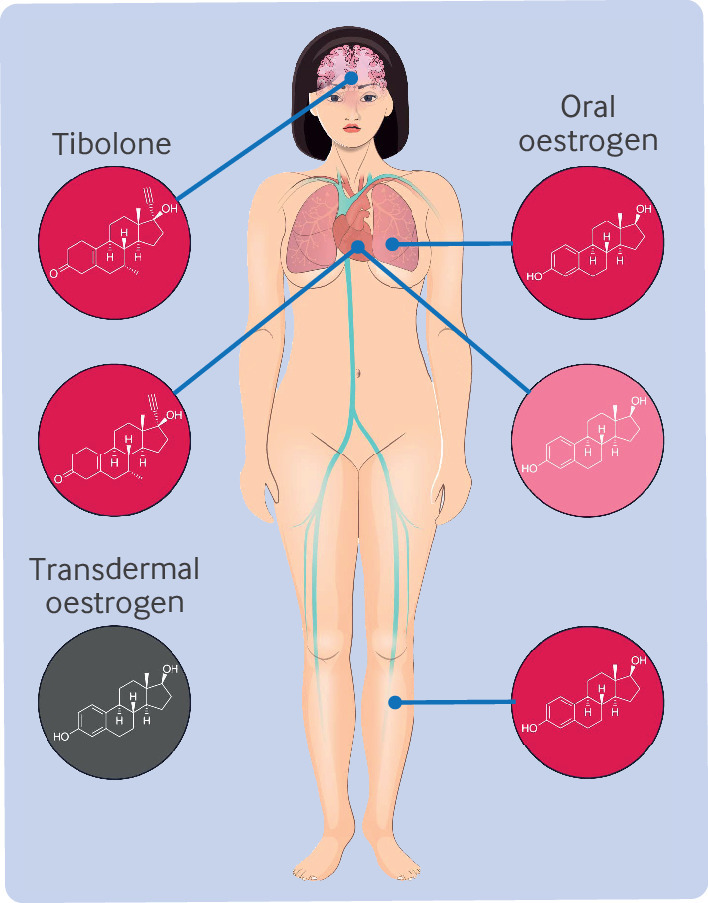
Illustration synthesising results of study. Tibolone was associated with increased risk of arterial thrombotic events, including ischaemic heart disease, myocardial infarction, and cerebral infarction, but not venous thromboembolism. Increased risk of venous thromboembolism and a small increased risk of ischaemic heart disease were found with use of oral continuously combined contemporary menopausal hormone therapy. No strong evidence was found that transdermal oestrogen increases risk for any diseases studied. Red=strong/intermediate effect; pink=small effect; grey=no effect detected

In the disease specific analysis, the use of oral combined continuous menopausal hormone therapy or tibolone was associated with a higher risk of ischaemic heart disease (hazard ratio 1.27 (95% CI 1.01 to 1.60) and 1.76 (1.14 to 2.70), respectively). In addition, we observed an increased risk of cerebral infarction in association with the use of tibolone; compared with never users; the hazard ratio was 1.97 (1.02 to 3.78). No other menopausal hormone therapy product was significantly associated with an increased risk of ischaemic heart disease or cerebral infarction. Women using tibolone had an increased risk of myocardial infarction (hazard ratio 1.94, 95% CI 1.01 to 3.73). None of the other analysed menopausal hormone therapy products showed a significant association with risk of myocardial infarction. As in our intention-to-treat analysis, women using oral oestrogen combined with progestogen, in either continuous or sequential forms, had an increased risk of venous thromboembolism, with hazard ratios of 1.84 (95% CI 1.50 to 2.25) for continuous use and 2.45 (1.89 to 3.17) for sequential use. Additionally, we observed an increased risk of venous thromboembolism with the use of transdermal combined menopausal hormone therapy, with a hazard ratio of 1.67 (1.16 to 2.41) (fig 4; supplementary table S10). Notably, although associated with cardiovascular disease, tibolone was not linked to an increased risk of venous thromboembolism (hazard ratio 0.76, 95% CI 0.38 to 1.53). This indicates a strong heterogeneity between the different menopausal hormone therapies with regards to the diseases investigated (fig 5).

## Discussion

In this Swedish nationwide emulated target trial of menopausal hormone therapy use in women around menopause, we found that starting oral combined continuous therapy or tibolone was associated with an increased risk of cardiovascular disease within the first two years of initiation. In disease specific analyses, the oral combined continuous regimen was associated with increased risks of ischaemic heart disease and venous thromboembolism, and tibolone was linked to an increased risk of ischaemic heart disease, cerebral infarction, and myocardial infarction but not venous thromboembolism (fig 5). We found no effect of transdermal regimens on the risk of all causes of cardiovascular disease (myocardial infarction, cerebral infarction, or ischaemic heart disease). Combined continuous menopausal hormone therapy was the most common regimen, accounting for 37% of overall initiations. However, we observed a clear trend towards increased use of transdermal products and oral oestrogen combined with the levonorgestrel intrauterine system between 2007 and 2020. Also, the use of tibolone, which has not been approved for use in, for example, the US, decreased in Sweden between the same years. This drop is encouraging; however, in 2018, approximately 1000 women initiated tibolone, which is estimated to have caused one stroke or ischaemic heart disease event.

### Trends in menopausal hormone therapy use and cardiovascular disease

During the study period, we observed a decrease in the use of menopausal hormone therapy, followed by an increase starting from around 2016. In Sweden, use dropped after key studies were published in 2002-03,^19^ ^20^ ^50^ but it stabilised from 2010 to 2016, a trend also seen in Spain,^51^ Australia,^52^ Korea,^53^ and New Zealand.^54^ Usage rose again after 2017. Updated Swedish guidelines in 2019, further revised in 2021, recommend starting within 10 years of menopause and not after age 60, reflecting new insights into the benefits and risks of menopausal hormone therapy.^55^ This shift, widely discussed among gynaecologists and general practitioners before its official adoption, likely influenced prescribing habits early on. Additionally, we noted a significant decrease of more than 50% in the incidence of cardiovascular disease, particularly ischaemic heart disease. This pattern is noted in other Nordic countries but contrasts with recent reports from the US, where rates of cardiovascular disease are increasing.^56^ Disparities between the two settings, such as access to high quality healthcare among people with low socioeconomic status, severe mental illness or immigrant background, most likely contribute to these differing trends. The longstanding Nordic system of promoting equitable and universal healthcare of high quality likely underpins the declining trend in cardiovascular disease observed in these countries.^57^

### Comparison with other studies

We used an emulated target trial design, which mimics a clinical trial, to estimate the effect of contemporary menopausal hormone therapy on the risk of cardiovascular diseases. In contrast to the Women’s Health Initiative trial, the largest randomised, placebo controlled trial of combined oral menopausal hormone therapy performed to date, we included women who were within the age of menopausal transition (50-58 years), the age when most women start using menopausal hormone therapy. However, similar results were seen in our study and the Women’s Health Initiative trials. The principal Women’s Health Initiative results identified an increased risk of stroke (hazard ratio 1.41, with 212 events), myocardial infarction (1.32, with 229 events), and venous thromboembolic disease (2.11, with 218 events).^20^ Furthermore, in an age stratified follow-up investigation, an increased risk of coronary heart disease was observed in the 50-59 years age group (hazard ratio 1.27, with 37 events).^21^ In our per protocol analyses, we observed an increased risk of ischaemic heart disease (hazard ratio 1.27) and venous thromboembolism (1.84) with the use of combined continuous menopausal hormone therapy. On the contrary, we did not observe any excess risk of stroke among users of oral oestrogen plus progestin. Important differences exist between the Women’s Health Initiative and our study that can potentially explain this difference. The Women’s Health Initiative investigated only one type of menopausal hormone therapy, specifically conjugated equine oestrogens plus medroxyprogesterone acetate, which has not been in use in Sweden since 2005. Conjugated equine oestrogens, compared with oestradiol (the most commonly used oestrogen in menopausal hormone therapy in Sweden), has been associated with an increased risk of stroke.^58^ The higher risk of stroke associated with menopausal hormone therapy use was also seen in an observational study performed in the UK Biobank cohort, in which most women had started their use before 2005.^6^ Our findings indicate that contemporary combined oral menopausal hormone therapy carries a lower risk of stroke than do products used previously and those that have been investigated in most previous studies.

With regards to cerebral infarction, a more recent Danish observational study reported an association between the use of oral menopausal hormone therapy and increased risk of ischaemic stroke,^7^ with estimates more similar to our per protocol analysis. Like our study, the Danish study also stratified by regimen and observed an increased risk of ischaemic stroke for oral preparations including combined continuous therapy, unopposed oral oestrogen, and tibolone, with the highest estimates found for continuous combined menopausal hormone therapy (relative rate 1.36). We observed an increased risk only in association with the use of tibolone, with a somewhat higher estimate (hazard ratio 1.97). The differences are possibly attributable to the analysis of prevalent users, a longer follow-up time (average 7.9 years), and the inclusion of cerebral apoplexy (ICD10 code: I64) in the Danish study, which included approximately 15-20% haemorrhagic strokes.^59^

The increased risk of venous thromboembolism with the use of menopausal hormone therapy has been reported in several previous studies.^6^ ^60^ ^61^ ^62^ ^63^ Most of the studies did not distinguish between different types of menopausal hormone therapy and assessed the overall risks associated with all formulations. Two case-control studies distinguished between different types of menopausal hormone therapy and reported an increased risk with the use oral combined menopausal hormone therapy (odds ratio 1.55 for continuous and 1.88 for sequential), lower risk with the use of oral oestrogen only (odds ratio 1.40), and no risk with tibolone or transdermal only products.^60^ Similarly, the LIFT trial observed no increased risk of venous thromboembolism with the use of tibolone, but an increased risk of stroke was found (hazard ratio 2.19).^64^ The LIBERATE trial assessed the safety and efficacy of tibolone in patients with breast cancer, focusing on cardiovascular outcomes as a secondary endpoint.^65^ It found no significant difference in cardiovascular risk between the tibolone group (14 cases) and the placebo group (10 cases). However, the trial’s limited sample size (n=3100) precluded detailed analyses of venous thromboembolism, cerebrovascular disease, and coronary heart disease. Considering our findings and previous research, tibolone seems to affect these conditions differently, suggesting that the study may have overlooked potential risks associated with cerebrovascular and coronary events.

### Biological mechanisms

The increased risk of cardiovascular disease and venous thromboembolism associated with oral oestrogen, in contrast to transdermal oestrogen, can be attributed to the route specific effects. When taken orally, oestrogen undergoes first pass metabolism in the liver, where many coagulation factors are produced. Therefore, oral administration of oestrogen is thought to influence the production of coagulation factors, leading to a net increase in procoagulant factors.^66^ Transdermal oestrogen minimises these effects by largely bypassing hepatic metabolism, resulting in less pronounced alterations in coagulation factors.^67^ Tibolone, unlike oral oestrogen, does not seem to increase the risk of venous thromboembolism. This difference may be due to tibolone’s unique pharmacological profile, combining oestrogenic, progestogenic, and androgenic effects, whereby its androgenic properties potentially increase fibrinolytic activity, reducing clot risk. However, the mechanism by which tibolone increases the risk of arterial thrombosis, including cerebral and myocardial infarction, remains unclear. A possible factor is an increase in C reactive protein concentrations, which could promote atherosclerotic plaque instability, making the plaque more likely to rupture and lead to thrombotic events such as heart attacks or strokes.^68^

### Strengths and limitations of study

Our study’s strengths include use of national registers to obtain recent health data on a large population. Use of registry data enabled us to obtain extensive health information on all women living in Sweden without the risk of recall bias. Leveraging advances in causal inference methods applied to high quality, linkable national registries enabled us to avoid this bias often seen in observational studies. The extensive information in the drug register also allowed us to distinguish between different types of hormone therapies, including differences in administration, regimens, and combinations of hormones.

This study does, however, have some limitations. The absence of specific data on menopausal status means that our study included only women aged 50 and above. This decision aligns with existing research indicating that early menopause is associated with a heightened risk of cardiovascular disease, whereas women entering menopause after 50 have a comparatively lower risk.^69^ By setting this age threshold, our aim was to ensure that the cohort predominantly consisted of postmenopausal women, thereby minimising any bias stemming from the varied timing of menopause. As a result, the calculated risk estimates for menopausal hormone therapy in our analysis should reflect a more precise correlation with cardiovascular disease risk, unaffected by the potential overestimation that could arise from including younger premenopausal or early menopausal women.^69^ Secondly, we did not have information on other potentially important confounders, such as smoking and body mass index. However, we adjusted for education and geographical region. Having a lower level of education and residing in a rural area have been associated with a threefold and twofold increase in the risk of obesity, respectively, compared with people with higher education residing in an urban area.^70^ Thirdly, we cannot confirm whether women used or merely collected the medication they redeemed. This could lead to misclassification of exposure, and any potential hazardous effects of menopausal hormone therapy on cardiovascular risk would be attenuated towards the null. However, to mitigate the impact of this limitation, our study used two analytical approaches: an intention-to-treat analysis and a per protocol analysis. The intention-to-treat analysis considered all women who collected menopausal hormone therapy as users from their first collection, ensuring inclusion based on the intended treatment. The per protocol analysis refined assessment of menopausal hormone therapy use by censoring women at discontinuation—defined as not redeeming further prescriptions—and thus more accurately representing consistent users of menopausal hormone therapy and mitigating misclassification bias. Fourthly, the prescription of unopposed oral oestrogen to women with an intact uterus is not standard clinical practice in Sweden. This occurrence in our study might reflect misclassifications due to hysterectomies performed internationally or outside our data collection period or the undocumented use of intrauterine devices or bioidentical progestogens. However, it could also indicate that some clinicians are prescribing unopposed oral oestrogen to women with an intact uterus. Fifthly, a crucial point in all registry based studies is the validity of the diagnostic codes. The diagnoses in the inpatient register are valid in 85-95% of most diagnoses. For myocardial infarction and stroke, the diagnoses were valid in more than 90% of patients; the figure for venous thromboembolism was 75%.^71^ Any diagnostic misclassification may have led to an underestimation of the risk among menopausal hormone therapy users. Sixthly, we could not use a pooled logistic regression model owing to computational limitations. As a result, the hazard ratios presented in our analysis may have a built-in selection bias. We also presented cumulative incidence curves and rates for the intention-to-treat analysis to mitigate this limitation. Lastly, our study focused on static treatment strategies. Although this approach provides valuable insights, it could be extended to meet patients’ characteristics more closely by considering dynamic strategies, which would allow treatment adjustments based on the development of contraindications over time.

### Meaning of study

A need has existed to re-evaluate the risk of cardiovascular disease associated with contemporary menopausal hormone therapy in women around the age of menopausal transition when menopausal hormone therapy is usually initiated. The feasibility of randomised trials to support decision making is particularly limited if the goal is to estimate effects in subgroups of women, such as those using different types of treatment. This study was specifically designed to avoid selection bias commonly seen in observational studies by emulating a target trial and to overcome the limitations of randomised trials by examining various types of menopausal hormone therapy products.

### Unanswered questions and future research

The study did not extend to the effects of specific progestins within these therapy formulations. Bioidentical progesterone, compared with synthetic progesterone (progestin), has been suggested to confer different risk profiles regarding cardiovascular disease.^72^ Hence, future research should investigate the potential various effects on the risk of cardiovascular disease based on different progestogens used in menopausal hormone therapy.

## What is already known on this topic

Starting conjugated equine oestrogen plus medroxyprogesterone acetate >10 years after menopause or at age >60 might increase the risk of heart disease, stroke, and venous thromboembolism
Research on the risk of cardiovascular disease associated with the use of contemporary menopausal hormone therapy during the menopausal transition age is lacking
Rigorous results from large populations are needed to fill this gap

## What this study adds

This large prospective population based cohort study included healthy women around the age of menopause
An increased risk of ischaemic heart disease and venous thromboembolism was found with the use of oral continuously combined contemporary menopausal hormone therapy
Tibolone was associated with an increased risk of arterial thrombotic events but not venous thromboembolism, highlighting the varying effects of different hormones on cardiovascular disease

## Data Availability

The analyses were based on data from different registries (Statistics Sweden and the National Board of Health and Welfare), and the data can be made available on reasonable request to each of the registry managers after an ethical approval from the Swedish Ethical Review Authority. The underlying code will be freely available at https://github.com/theresejohansson/CVD_MHT.

## Web extra

Extra material supplied by authors

Web appendix: Supplementary materials

## Notes

Contributors: TJ was involved in data curation, conceptualisation, software, visualisation, and formal analyses and wrote the first draft of the manuscript. TK was involved in study supervision. DS was involved in software. AS and JRC were involved in conceptualisation. ÅJ was involved in funding acquisition, study supervision, resources, and conceptualisation. All authors critically reviewed and edited the manuscript, approved the final version for submission, and agreed to be accountable for all aspects of this work. TJ is the guarantor. The corresponding author attests that all listed authors meet authorship criteria and that no others meeting the criteria have been omitted.

Transparency: The lead author (the manuscript’s guarantor) affirms that the manuscript is an honest, accurate, and transparent account of the study being reported, that no important aspects of the study have been omitted, and that any discrepancies from the study as planned (and, if relevant, registered) have been explained.

Dissemination to participants and related patient and public communities: Findings from this study will be included and considered in clinical recommendations of menopausal hormone therapy use in Sweden. To reach the public, results from this study will be shared with the public via a press release alongside the publication of this manuscript. Social media (eg, X and LinkedIn) will be used to draw attention to the work and stimulate debate about its findings.

Provenance and peer review: Not commissioned; externally peer-reviewed.
