# Supplementary material for: Contemporary menopausal hormone therapy and risk of cardiovascular disease: Swedish nationwide register based emulated target trial
Source: BMJ. 2024 Nov 27;387:e078784. doi: 10.1136/bmj-2023-078784 (PMC11600536; doi:10.1136/bmj-2023-078784)
Supplement: Supplementary file 1 — Web appendix: Supplementary materials [file joht078784.ww1.pdf]

Supplementary material

Contemporary menopausal hormone therapy and risk of cardiovascular disease - a Swedish nationwide register-based emulated target trial

Supplementary methods

Covariates

Fine & Gray subdistribution model

Inverse probability weighting

Figures

Figure S1. Directed acyclic graph to identify potential confounders

Figure S2. Inverse probability weighting adjusted cumulative incidence curves

Supplementary tables

Table S1. Eligibility criteria of study population

Table S2. Number of participants, initiators, and cases in each non-randomised trial

Table S3. Study outcomes

Table S4. Treatment strategies

Table S5. Adjustment variables

Table S6. Adjusted incidence rates within specific time frames

Table S7. Intention-to-treat analyses with unadjusted hazard ratios, adjusted incidence rates and adjusted hazard ratios for cardiovascular disease and cause-specific cardiovascular disease with the use of menopausal hormone therapy

Table S8. Intention-to-treat analyses with adjusted subdistribution hazard ratios for cardiovascular disease and cause-specific hazard ratios with the use of menopausal hormone therapy.

Table S9. Intention-to-treat analyses with adjusted hazard ratios for cardiovascular disease and cause-specific hazard ratios with use of menopausal hormone therapy with study period 2011 to 2020.

Table S10. Per-protocol analyses with unadjusted hazard ratios, adjusted incidence rates and adjusted hazard ratios for cardiovascular disease and cause-specific hazard ratios with the use of menopausal hormone therapy.

Supplementary methods

Covariates

The study classified the participants into five groups based on their level of education and length of schooling. The first group consisted of women who had completed elementary school (9-10 years of schooling), while the second group comprised women who had completed high school (2-3 years after elementary school). The third group consisted of women with higher education (2-6 years after high school), while the fourth group included women with research education. The fifth category included women for whom no information about their education was available. The participants were further classified based on their place of residence into four categories that reflected the differences between rural and urban regions. The first category included women living in Stockholm, Uppsala, Södermanland, and Östergötland. The second category included women from Jönköping, Kronobergs län, Kalmar, Gotland, and Blekinge. The third category included women from Skåne, Västra götaland, Värmland, Örebro, and Västmanland, while the fourth category included women from Dalarna, Gävleborgslän, Västernorrland, Jämtland, Västerbotten, and Norrbotten. The study also accounted for ancestral origin depending on the region of birth. Eight strata were applied for this purpose. The first group consisted of women born in the Nordic region (including Sweden), while the second group included women born in Europe (excluding the Nordics). The third group included women born in Africa, while the fourth group comprised women born in Asia. The fifth group consisted of women born in North America, while the sixth group included women born in South America. The seventh group comprised women born in Oceania, and the eighth group consisted of women born in the former Union Republics of the Soviet Union. Adjustments were made for predisposed diseases including hypertension, heart disease, and diabetes, based on medication use. Henceforth, this time-varying covariate will be denoted “medication status”. In the intention-to-treat analysis, we included baseline information. In the per-protocol analysis, we adjusted for each participant's current medication status, as changes in medication could influence exposure level.

Fine & Gray subdistribution models

In our main analysis, we calculated cause-specific hazards, which represent the rate of occurrence of the event of interest at a given time for individuals who have not yet experienced any event. This means we only consider subjects who have not yet experienced the event of interest or any competing risks. For sensitivity analysis, we also used the Fine-Gray regression model based on the subdistribution hazard function[1]. The subdistribution for a specific outcome is the rate of experiencing that particular outcome at a given time, given that the individual has not yet experienced failure from that outcome. For instance, if we are interested in the subdistribution hazard for cerebral infarction, individuals who died before experiencing a cerebral infarction are still considered at risk for cerebral infarction. In contrast, for our cause-specific hazard, individuals who die from other causes are no longer considered to be in the risk set. To calculate the subdistribution hazards, we used the finegray function in the R survival package, where death, censoring and the event of interest were set as separate outcomes compared to the event of interest (censored = 0, event of interest = 1, death or emigration = 2).

Inverse probability weighting

In our intention-to-treat analysis, we used inverse probability of treatment weights (IPTW) to adjust for baseline confounding. We estimated stabilised treatment weights as the marginal probability of treatment divided by the probability of treatment conditional on measured baseline covariates. We used logistic regression to estimate our weights and then truncated them at the 99th and 1st percentiles to prevent outliers from influencing the analysis. Finally, to estimate the association between menopausal hormone therapy and cardiovascular disease, we estimated hazard ratios using Cox regression hazard models weighted by the IPTW.

In our per-protocol analysis, we used inverse probability of treatment weights to adjust for baseline confounding and time-varying confounding. We calculated the stabilised treatment weights as the marginal probability of treatment given the measured baseline covariates divided by the probability of treatment given the measured baseline covariates and the time-varying covariate "predisposed diseases or disorders for cardiovascular disease", as briefly described above. We used Cox regression to estimate our stabilised (truncated at the 99th and 1st percentiles) weights.

In our per-protocol analysis, we excluded participants who deviated from the study protocol. However, this type of artificial censoring can introduce bias because those who deviate from the protocol may differ from those who follow it, and this difference may be related to the outcome. Additionally, loss to follow-up, such as when participants emigrate, can also lead to selection bias. Another example is that patients with poor health status may be more likely to discontinue menopausal hormone therapy before a cardiovascular event, which may lead to an overestimated survival probability of users of menopausal hormone therapy. To address the potential bias that may arise from informative censoring, we utilised inverse probability of censoring weights (IPCW)[2]. We applied the weighting to up-weight those remaining in the study who have similar characteristics to those who were censored. To implement IPCW, we utilised a time-varying Cox proportional hazards model. In this approach, a new row of data for an individual was created only when there was a change in any of the time-varying covariates for that person. We calculated the IPCW based on previous exposure and patient characteristics related to censoring. Finally, we multiplied the IPTW with the IPCW to obtain a single weight for each individual and included this weight in the Cox regression model.

Inverse probability weights for point-treatment were estimated using the ipwpoint function in the 'ipw' package, while inverse probability weights for censoring were estimated using the ipwtm function in the same package[3].

The summary statistics of the stabilised IPTWs were as followed: Mean: 0.9999, Min: 0.1408, Max: 6.5217, Standard deviation: 0.0162

Figures

A

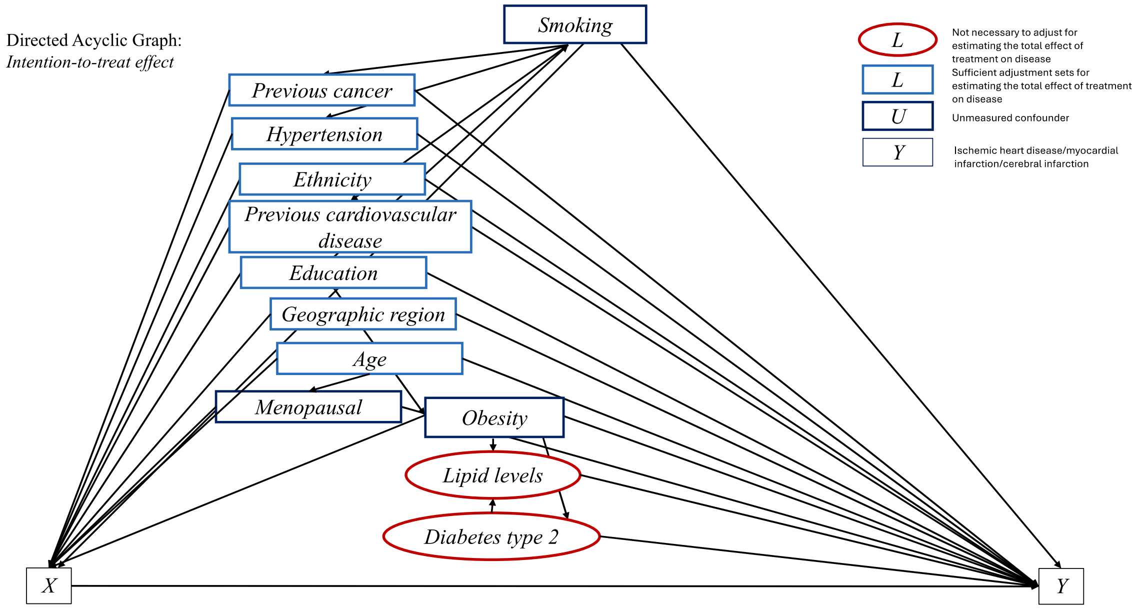

B

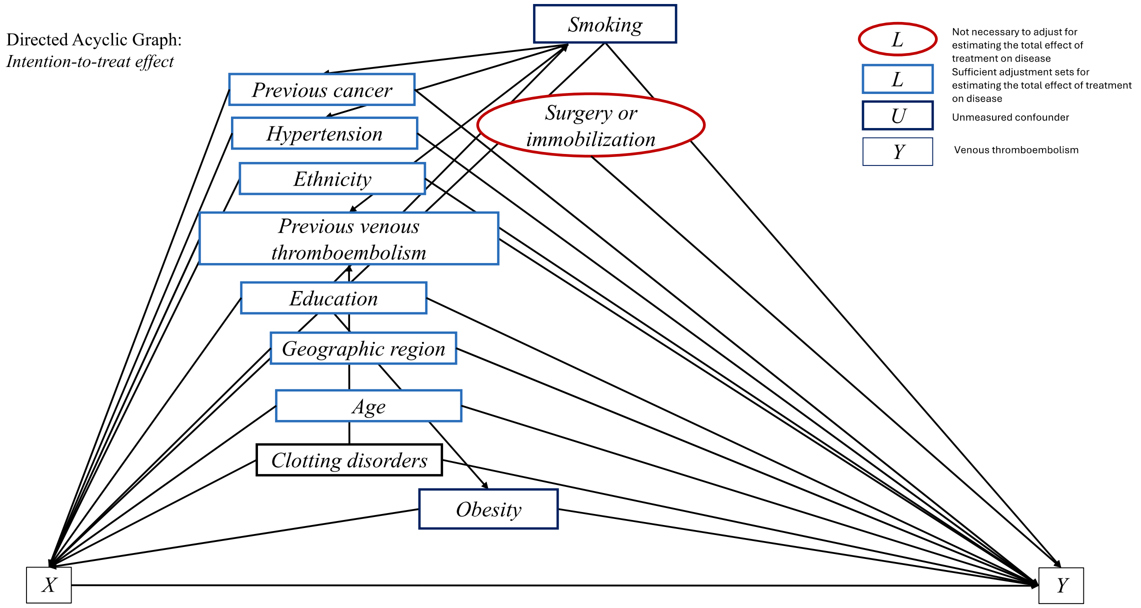

Figure S1A-B*. Directed Acyclic Graph (DAG) to identify potential confounders. X = Exposure. Y = Outcome.* ***A****: DAG to identify potential confounders for the relationship between menopausal hormone therapy and cardiovascular disease (ischemic heart disease, myocardial infarction & stroke).* ***B****: DAG to identify potential confounders for the relationship between menopausal hormone therapy and venous thromboembolism. Note: The light blue boxes indicate the adjustment set included in the models and reported in the Tables.*

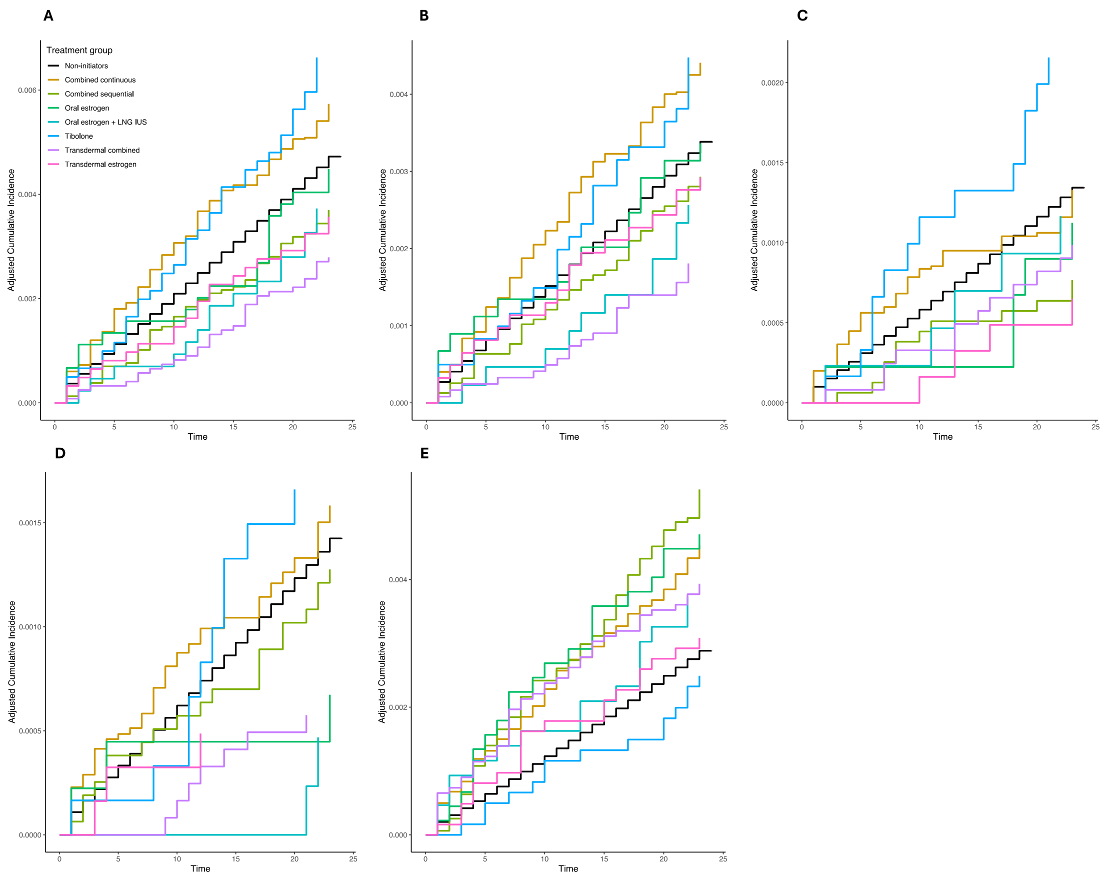

Figure S2. *Inverse probability weighting adjusted cumulative incidence curves for the intention-to-treat analysis. A: Cardiovascular disease (includes ischemic heart disease, myocardial and cerebral infarction), B: Ischemic heart disease, C: Cerebral infarction, D: Myocardial infarction, E: Venous thromboembolism*

Table S1. Eligibility criteria of study population

**Eligibility criteria**	**Response**	**Data source**	**Period of data availability**			
Target population	Healthy women between 50-58 years of age	Total Population Register	1961-2020			
Time period study subjects were identified	From 1st of July 2007 until December 2020	Total Population Register	1961-2020			
Sex	Females	Total Population Register	1961-2020			
**Exclusion criteria**		**Data source**	**Period of data availability**	**ICD8**	**ICD9**	**ICD10**
Cerebral infarction		The National Patient Register & The Cause of Death register	1987-2020, 1952-2020	433, 434	433, 434, 436, 437	I63
Non-traumatic intracerebral haemorrhage		The National Patient Register & The Cause of Death register	1987-2020, 1952-2020	431	431	I61
Non-traumatic subarachnoid haemorrhage		The National Patient Register & The Cause of Death register	1987-2020, 1952-2020	430	430	I60
Intracranial haemorrhage		The National Patient Register & The Cause of Death register	1987-2020, 1952-2020	431	431	I62

Cerebral apoplexy	The National Patient Register & The Cause of Death register	1987-2020, 1952-2020	436	436	I64
Acute myocardial infarction	The National Patient Register & The Cause of Death register	1987-2020, 1952-2020	410	410	I21-I22
Ischemic heart disease	The National Patient Register & The Cause of Death register	1987-2020, 1952-2020	411-414	411-414, 429	I20, I21, I22, I24, I25
Pulmonary embolism	The National Patient Register & The Cause of Death register	1987-2020, 1952-2020	450	415	I26
Thrombosis of intracranial vein	The National Patient Register & The Cause of Death register	1987-2020, 1952-2020	438	437	I67.6
Thrombophlebitis	The National Patient Register & The Cause of Death register	1987-2020, 1952-2020	451	451	I80-I80.9
Portal thrombosis	The National Patient Register & The Cause of Death register	1987-2020, 1952-2020	452	452	I81
Venous embolism and thrombosis	The National Patient Register & The Cause of Death register	1987-2020, 1952-2020	453	453	I82

Breast cancer	The National Patient Register, The Cause of Death register & The Cancer Register	1987-2020, 1952-2020, 1958-2020	174	174	C50
Cervical cancer	The National Patient Register, The Cause of Death register & The Cancer Register	1987-2020, 1952-2020, 1958-2020	I80	I80	C53
Endometrial cancer	The National Patient Register, The Cause of Death register & The Cancer Register	1987-2020, 1952-2020, 1958-2020	182	182	C54
Cancer of womb without specification	The National Patient Register, The Cause of Death register & The Cancer Register	1987-2020, 1952-2020, 1958-2020	179	179	C55
Ovarian cancer	The National Patient Register, The Cause of Death register & The Cancer Register	1987-2020, 1952-2020, 1958-2020	183	183	C56
Tubal cancer, non-specified cancer in female genitals	The National Patient Register, The Cause of Death register & The Cancer Register	1987-2020, 1952-2020, 1958-2020	183, 184	183, 184	C57
Colon cancer	The National Patient Register, The Cause of Death register & The Cancer	1987-2020, 1952-2020, 1958-2020	153	163	C18

	Register				
Rectum cancer	The National Patient Register, The Cause of Death register & The Cancer Register	1987-2020, 1952-2020, 1958-2020	154	154	C19
Malignant lymphoma of Hodgkin lymphoma	The National Patient Register, The Cause of Death register & The Cancer Register	1987-2020, 1952-2020, 1958-2020	201	201	C81
Malignant lymphoma non Hodgkin lymphoma, follicular type	The National Patient Register, The Cause of Death register & The Cancer Register	1987-2020, 1952-2020, 1958-2020	200	200	C82
Malignant lymphoma non Hodgkin lymphoma, diffuse type	The National Patient Register, The Cause of Death register & The Cancer Register	1987-2020, 1952-2020, 1958-2020	200	200	C83
T-cell malignant lymphoma in skin or systemic	The National Patient Register, The Cause of Death register & The Cancer Register	1987-2020, 1952-2020, 1958-2020	202	202	C84
Malignant lymphoma non hodgkin, non-specified	The National Patient Register, The Cause of Death register & The Cancer Register	1987-2020, 1952-2020, 1958-2020	200	200	C85

Malignant immunoproliferative disease	The National Patient Register, The Cause of Death register & The Cancer Register	1987-2020, 1952-2020, 1958-2020	200	200	C88
Multiple myeloma and malignant plasma and cell neoplasms	The National Patient Register, The Cause of Death register & The Cancer Register	1987-2020, 1952-2020, 1958-2020	203	203	C90
Lymphoid leukemia	The National Patient Register, The Cause of Death register & The Cancer Register	1987-2020, 1952-2020, 1958-2020	204	204	C91
Myeloid leukemia	The National Patient Register, The Cause of Death register & The Cancer Register	1987-2020, 1952-2020, 1958-2020	205	205	C92
Other leukemias of specified cell type	The National Patient Register, The Cause of Death register & The Cancer Register	1987-2020, 1952-2020, 1958-2020	207	207	C94
Leukemias of unspecified cell type	The National Patient Register, The Cause of Death register & The Cancer Register	1987-2020, 1952-2020, 1958-2020	208	208	C95
Other and unspecified malignant neoplasms of lymphoid, hematopoeitic and related tissues	The National Patient Register, The Cause of Death register & The Cancer	1987-2020, 1952-2020, 1958-2020	202	202	C96

	Register				
**Surgical procedures excluded**			**Surgical codes**		
Hysterectomy	The National Patient Register	1996-2020	LCD00-97		
Unilateral oophorectomy (two occurrences)	The National Patient Register	1996-2020	LAE10/11		
Unilateral salpingoophorectomy (two occurrences)	The National Patient Register	1996-2020	LAF00/01		
Bilateral oophorectomy	The National Patient Register	1996-2020	LAE20/21		
Salpingoophorectomy bilateral	The National Patient Register	1996-2020	LAF10/11		
**Previous medication use excluded**	**Data source**	**Period of data availability**	**Codes**		
No previous use of systemic menopausal hormone therapy in the past 2 years	The Prescribed Drug Register	2005-2020	G03CA03, G03CA04, G03CA57, G03CC07, G03FA01, G03FA12, G03FA14, G03FA15, G03FA17, G03FB05, G03FB06, G03CX01, G03DA, G03DC, G03BA03		

Table S2. Number of participants, initiators and events in each non-randomised trial

Trial number	Calendar month	Before exclusion	Previous MHT use	Previous CVD	Previous cancer	Previous surgery	Eligible study participants	CVD events	Initiators	Events in initiators
1	Jul-07	497709	70898	22553	50550	32193	350871	3147	331	8
2	Aug-07	502242	70734	23158	51074	32622	354535	3172	646	9
3	Sep-07	507025	70667	23574	51697	33101	358113	3161	674	9
4	Oct-07	511629	70686	23961	52365	33642	361412	3169	684	9
5	Nov-07	516016	70791	24371	53054	34199	364409	3193	642	6
6	Dec-07	520480	70938	24777	53691	34748	367468	3209	452	5
7	Jan-08	518335	70171	24809	53729	35007	366754	3130	610	6
8	Feb-08	517725	69431	24859	53779	35279	366487	3133	615	7
9	Mar-08	517285	68923	24870	53758	35565	366322	3112	504	5
10	Apr-08	516910	68227	24924	53799	35802	366336	3123	703	7
11	May-08	516682	67925	24937	53845	36076	366163	3125	603	6
12	Jun-08	516203	67745	24968	53910	36351	365794	3105	531	2
13	Jul-08	515687	66922	24976	53911	36662	365535	3092	309	2
14	Aug-08	515175	66274	24971	53842	36889	365397	3098	462	4
15	Sep-08	514905	65626	25029	53799	37151	365459	3090	679	15
16	Oct-08	514688	65250	25048	53859	37466	365215	3077	568	7
17	Nov-08	514620	64686	25129	53944	37772	365274	3073	490	5
18	Dec-08	514444	64131	25143	54063	38011	365276	3097	416	2
19	Jan-09	512434	63546	25210	54118	38271	364460	2997	439	1
20	Feb-09	512098	62773	25244	54127	38513	364543	2980	425	5
21	Mar-09	511931	62062	25285	54134	38771	364776	2955	546	4
22	Apr-09	511662	61482	25327	54115	39108	364747	2967	549	5
23	May-09	511734	60967	25374	54189	39493	364891	2948	474	5

24	Jun-09	511688	60412	25399	54228	39777	365099	2989	535	4
25	Jul-09	511705	60004	25431	54315	40075	365104	2954	314	3
26	Aug-09	511705	59394	25393	54299	40295	365437	2960	482	3
27	Sep-09	511759	58883	25449	54310	40526	365657	2980	672	11
28	Oct-09	511600	58621	25525	54351	40833	365373	2984	516	5
29	Nov-09	511335	58142	25582	54407	41110	365214	3013	515	7
30	Dec-09	511210	57648	25592	54456	41396	365274	3040	404	2
31	Jan-10	509332	57250	25639	54493	41698	364364	2992	454	4
32	Feb-10	509399	56646	25725	54507	42000	364611	3023	485	6
33	Mar-10	509480	56131	25714	54483	42348	364870	3025	632	13
34	Apr-10	509725	55946	25772	54543	42672	364941	3023	563	6
35	May-10	509601	55477	25799	54510	42916	365029	3023	599	4
36	Jun-10	509451	55112	25842	54561	43268	364832	3075	668	2
37	Jul-10	509498	54767	25904	54569	43575	364856	3068	274	2
38	Aug-10	509528	54224	25935	54577	43786	365161	3053	542	4
39	Sep-10	509831	53926	26009	54582	44058	365430	3064	607	7
40	Oct-10	509935	53709	26071	54666	44341	365410	3061	634	6
41	Nov-10	510023	53528	26176	54768	44623	365327	3056	543	5
42	Dec-10	509917	53277	26228	54845	44908	365112	3055	415	9
43	Jan-11	508355	52900	26275	54928	45191	364440	2997	478	11
44	Feb-11	508276	52560	26326	54948	45483	364425	3019	521	3
45	Mar-11	508579	52408	26301	54928	45801	364655	3001	576	4
46	Apr-11	508719	52136	26373	54934	46099	364702	2969	571	7
47	May-11	508630	51960	26340	54889	46318	364655	3009	677	5
48	Jun-11	508593	51866	26425	54946	46576	364360	2979	594	3
49	Jul-11	508600	51730	26455	54899	46838	364309	2975	322	2

50	Aug-11	508663	51365	26477	54866	47037	364537	2982	597	6
51	Sep-11	508553	51168	26562	54824	47350	364285	2963	652	9
52	Oct-11	508523	51108	26605	54899	47655	363988	2969	591	4
53	Nov-11	508621	51061	26733	55001	47919	363742	2966	535	4
54	Dec-11	508512	50902	26844	55082	48238	363434	2944	433	5
55	Jan-12	506875	50737	26896	55094	48446	362626	2881	483	5
56	Feb-12	507168	50557	27038	55198	48783	362617	2862	477	3
57	Mar-12	507279	50450	27078	55215	49075	362601	2851	558	4
58	Apr-12	507723	50281	27136	55325	49365	362878	2855	507	2
59	May-12	507794	50188	27179	55364	49587	362772	2815	603	3
60	Jun-12	507918	50163	27286	55489	49872	362547	2816	546	7
61	Jul-12	507840	50061	27318	55469	50110	362374	2794	375	2
62	Aug-12	507798	49869	27354	55444	50234	362356	2800	542	2
63	Sep-12	507953	49739	27374	55469	50444	362372	2801	619	5
64	Oct-12	508350	49705	27416	55530	50770	362450	2802	619	9
65	Nov-12	508593	49738	27505	55627	51073	362350	2803	596	1
66	Dec-12	508612	49743	27544	55705	51362	362080	2826	354	6
67	Jan-13	507098	49539	27586	55777	51642	361443	2784	494	1
68	Feb-13	507290	49461	27673	55829	51920	361412	2792	472	3
69	Mar-13	507998	49404	27695	55859	52290	361799	2802	505	4
70	Apr-13	508658	49339	27763	55873	52647	362117	2815	539	7
71	May-13	509013	49323	27813	55873	52922	362210	2798	561	5
72	Jun-13	509424	49339	27892	55954	53203	362331	2802	532	4
73	Jul-13	509891	49332	27908	55984	53478	362542	2832	283	5
74	Aug-13	510210	49107	27952	55973	53695	362847	2810	440	1
75	Sep-13	510772	48932	28005	55971	54024	363264	2807	602	3

76	Oct-13	511297	48980	28081	56110	54369	363357	2822	609	5
77	Nov-13	511780	49089	28189	56240	54716	363291	2800	554	8
78	Dec-13	512227	49118	28226	56371	55033	363309	2764	457	5
79	Jan-14	510824	49123	28290	56352	55277	362595	2708	512	1
80	Feb-14	511846	49111	28379	56464	55654	363177	2749	543	5
81	Mar-14	512590	49129	28449	56554	55987	363512	2750	634	3
82	Apr-14	513646	49263	28542	56677	56358	364034	2736	589	8
83	May-14	514589	49391	28516	56712	56671	364620	2750	578	5
84	Jun-14	515221	49394	28608	56772	56975	364872	2738	598	4
85	Jul-14	515815	49571	28618	56833	57229	365064	2741	322	3
86	Aug-14	516122	49310	28632	56815	57390	365469	2736	485	5
87	Sep-14	517030	49291	28702	56903	57659	366040	2748	632	1
88	Oct-14	517871	49376	28795	57031	57971	366400	2748	623	2
89	Nov-14	518697	49518	28872	57123	58283	366770	2775	548	6
90	Dec-14	519674	49653	28981	57266	58608	367182	2782	457	3
91	Jan-15	518431	49652	29062	57285	58906	366475	2746	465	3
92	Feb-15	519187	49604	29131	57333	59171	367005	2741	498	2
93	Mar-15	520168	49653	29177	57400	59501	367661	2755	578	4
94	Apr-15	521151	49813	29193	57498	59839	368188	2790	567	1
95	May-15	522098	49879	29255	57579	60136	368778	2787	576	2
96	Jun-15	522827	50007	29381	57644	60429	369065	2767	617	4
97	Jul-15	523315	50158	29479	57734	60708	369102	2723	331	3
98	Aug-15	523890	50059	29521	57767	60859	369605	2739	581	4
99	Sep-15	524569	50199	29562	57676	61060	370064	2752	690	6
100	Oct-15	525377	50404	29661	57816	61322	370348	2729	655	6
101	Nov-15	525899	50656	29730	57917	61620	370330	2743	615	1

102	Dec-15	526301	50873	29769	58005	61877	370312	2743	472	6
103	Jan-16	525138	50856	29777	58064	62076	369860	2691	448	4
104	Feb-16	526021	50771	29885	58102	62342	370433	2701	540	3
105	Mar-16	526778	50808	29931	58085	62590	370975	2688	553	5
106	Apr-16	527616	50827	29978	58237	62811	371486	2695	616	1
107	May-16	528283	50991	30038	58296	63093	371787	2684	578	6
108	Jun-16	528453	50938	30104	58277	63254	371869	2691	551	4
109	Jul-16	528895	50917	30132	58308	63398	372205	2667	350	3
110	Aug-16	529744	50792	30165	58277	63472	373031	2674	552	5
111	Sep-16	530521	50779	30196	58313	63652	373571	2689	726	5
112	Oct-16	531310	51012	30264	58376	63880	373869	2660	632	4
113	Nov-16	531914	51098	30357	58413	64137	374117	2678	494	10
114	Dec-16	532441	51045	30430	58561	64371	374382	2675	432	4
115	Jan-17	531168	50993	30495	58656	64588	373776	2633	460	10
116	Feb-17	531986	50950	30594	58785	64786	374316	2670	496	8
117	Mar-17	533079	50997	30630	58881	65048	375086	2685	655	4
118	Apr-17	533923	51096	30662	58995	65285	375581	2653	553	3
119	May-17	534688	51140	30739	59094	65503	376034	2641	600	6
120	Jun-17	535646	51282	30809	59232	65695	376560	2642	631	4
121	Jul-17	536471	51439	30871	59382	65855	376942	2614	337	3
122	Aug-17	537193	51339	30875	59427	65905	377643	2616	651	4
123	Sep-17	537611	51481	30927	59524	65995	377794	2617	668	9
124	Oct-17	538209	51643	30963	59660	66188	378016	2615	693	9
125	Nov-17	538500	51837	31059	59728	66306	377998	2611	665	4
126	Dec-17	538715	52059	31133	59805	66443	377878	2607	518	4
127	Jan-18	539444	52128	31145	59832	66551	379173	2594	576	3

128	Feb-18	540303	52285	31277	60018	66852	379448	2598	531	6
129	Mar-18	540875	52259	31309	60064	67018	379872	2600	618	2
130	Apr-18	541585	52496	31357	60194	67167	380225	2612	667	7
131	May-18	541881	52703	31375	60251	67201	380298	2599	692	5
132	Jun-18	542150	52592	31419	60275	67289	380250	2601	564	5
133	Jul-18	542317	52583	31422	60324	67407	380331	2607	385	4
134	Aug-18	542843	52672	31444	60354	67413	380836	2603	635	8
135	Sep-18	543386	52903	31526	60432	67489	381131	2598	731	5
136	Oct-18	543960	54076	31580	60579	67624	381237	2615	1411	10
137	Nov-18	544303	55915	31722	60749	67755	380401	2602	1897	12
138	Dec-18	544517	56731	31797	60954	67878	378943	2616	1160	6

Previous menopausal hormone therapy use is defined as previous use within the last two years (the washout period). Previous cardiovascular disease, surgery and cancer includes all diagnoses from the patient registry, cancer registry and cause of death registry which dates back to 1968. CVD = Cardiovascular disease. MHT = Menopausal hormone therapy.

Table S3. Study outcomes obtained from the Swedish National Patient Registry and Cause of Death Registry

Outcomes	Variable Values
First incident of composite cardiovascular disease	ICD-10 codes: I20, I21, I22, I24, I25, I63
Ischemic heart disease	ICD-10 codes: I20, I21, I22, I24, I25
Cerebral infarction	ICD-10 code: I63
Myocardial infarction	ICD-10 codes: I21, I22
Venous thromboembolism	ICD-10 codes: I26, I80, I81, I82

ICD-10 code is an abbreviation for International Classification of Disease Tenth Revision. ICD-10 code I20 = Angina pectoris, I21 = Acute myocardial infarction, I22 = Subsequent myocardial infarction, I24 = Other acute ischemic heart diseases, I25 = Chronic ischemic heart disease (includes conditions like angina pectoris, atherosclerotic cardiovascular disease, and all chronic forms of heart disease resulting from reduced blood flow to the heart muscle), I26 = Pulmonary embolism, I63 = Cerebral infarction, I80 = Phlebitis and thrombophlebitis, I81 = Portal vein thrombosis.

Table S4. Treatment strategies

Treatment Strategies	Response	Variable Values
Strategy 1	Initiators of oral combined continuous products<br><br>1-2 mg estradiol/1-2 mg estriol/0.625 mg conjugated equine estrogen<br><br>0.1-5 mg norethisterone/2 mg drospirenone/1-10 mg medroxyprogesterone/2.5-5.0 mg dydrogesterone/25-400 mg progesterone/ Gel 8% progesterone	G03FA01, G03FA12, G03FA14, G03FA15, G03FA17 or G03CA03/04 + G03DC/DA
Strategy 2	Initiators of oral combined sequential products<br><br>1-2 mg estradiol/1-2 mg estriol with:<br><br>1-5 mg norethisterone / 5-20 mg medroxyprogesterone / 10 mg dydrogesterone/25-400 mg progesterone/Gel 8% progesterone	G03FB05, G03FB06, G03FB08, or G03CA03/04 + G03DC/DA
Strategy 3	Initiators of unopposed oral estrogen<br><br>1-2 mg estradiol/1-2 mg estriol	G03CA03, G03CA04
Strategy 4	Initiators of oral estrogen + levonorgestrel intrauterine system<br><br>1-2 mg estradiol/1-2 mg estriol with:<br><br>13.5-52.0 mg levonorgestrel	G03CA03/04 + G02BA03
Strategy 5	Initiators of tibolone	G03CX01

	2.5 mg tibolone	
Strategy 6	Initiators of transdermal combined<br><br>0.025 – 1.53 mg estradiol with:<br><br>5 mg norethisterone/5-10 mg medroxyprogesterone/25-400 mg progesterone/Gel 8% progesterone	G03FA01, G03FB05, G03CA03 + G03DA/DC/G03BA03
Strategy 7	Initiators of transdermal unopposed estrogen<br><br>0.025 – 1.53 mg estradiol	G03CA03
Strategy 8	Non-initiators of menopausal hormone therapy	No filled prescriptions of any of the above Anatomical Therapeutical Chemical codes

Table S5. Adjustment variables

Adjustment Variables	Data source	Data availability	Variable Values
Age at baseline	Total Population Register	1961-2020	Date of Birth
Ancestral origin	Total Population Register	1961-2020	UtlSvBakg
Highest achieved education	The Education Register	1961-2020	SUN2000Niva
Residence of living	Total Population Register	1961-2020	Lan
Predisposing disease and disorders: Diabetes, heart disease and hypertension.	The Prescribed Drug Register	2005-2020	Medications for diabetes include the Anatomical Therapeutical Codes A10A and A10B, for heart disease C01, C08, C09 and C10, for hypertension C02, C03, C04, C07.
Trial month			
Calendar year of trial			

Note: UtlSvBakg is an abbreviation in Swedish and stands for Foreigner-Swedish-Background and was used to define the study participants ancestral origin. Sun2000Niva is an abbreviation in Swedish, SUN stands for Swedish education classification and was created in the end of 1990 to define the level of education. Lan stands for municipality and was used to define the study participants area of residence (urban or rural).

Table S6. Adjusted incidence rates within specific time frames for the intention-to-treat analysis.

	Adjusted incidence rates within restricted follow-up (No of events/1 000 person-years)*				
Type of hormonal contraception	4 months (95% CI)	8 months (95% CI)	12 months	18 months	24 months
**Cardiovascular disease**					
Non-initiators	2.56 (2.50 to 2.63)	2.53 (2.48 to 2.67)	2.47 (2.43 to 2.50)	2.45 (2.43 to 2.48)	2.17 (2.15 to 2.20)
Combined continuous	3.34 (2.39 to 4.68)	3.29 (2.58 to 4.19)	3.08 (2.51 to 3.78)	2.58 (2.15 to 3.10)	2.42 (2.06 to 2.86)
Combined sequential	2.14 (1.19 to 3.88)	2.19 (1.45 to 3.34)	2.09 (1.47 to 2.97)	2.01 (1.49 to 2.70)	2.00 (1.55 to 2.59)
Oral estrogen	3.45 (1.55 to 7.67)	2.06 (0.98 to 4.31)	1.78 (0.93 to 3.42)	2.15 (1.31 to 3.50)	2.03 (1.31 to 3.14)
Oral estrogen plus LNG IUS	1.63 (0.41 to 6.51)	1.24 (0.40 to 3.84)	1.66 (0.75 to 3.70)	1.87 (1.01 to 3.48)	2.26 (1.39 to 3.69)
Tibolone	2.90 (1.30 to 6.46)	3.18 (1.84 to 5.47)	3.28 (2.11 to 5.08)	3.21 (2.23 to 4.62)	3.33 (2.44 to 4.54)
Transdermal combined	1.04 (0.39 to 2.80)	1.06 (0.53 to 2.13)	1.16 (0.67 to 2.00)	1.57 (1.07 to 2.30)	1.54 (1.10 to 2.16)
Transdermal unopposed estrogen	2.54 (1.06 to 6.12)	1.83 (0.87 to 3.85)	2.12 (1.21 to 3.74)	2.04 (1.27 to 3.28)	1.99 (1.31 to 3.03)
**Ischemic heart disease**					
Non-initiators	1.88 (1.83 to 1.94)	1.80 (1.76 to 1.84)	1.75 (1.72 to 1.78)	1.73 (1.61 to 1.66)	1.66 (1.54 to 1.59)
Combined continuous	2.16 (1.42 to 3.29)	2.40 (1.81 to 3.20)	2.26 (1.78 to 2.88)	1.98 (1.61 to 2.45)	1.86 (1.55 to 2.24)
Combined sequential	1.94 (1.04 to 3.61)	1.60 (0.98 to 2.61)	1.56 (1.03 to 2.34)	1.60 (1.15 to 2.23)	1.59 (1.19 to 2.13)
Oral estrogen	2.81 (1.17 to 6.76)	1.74 (0.78 to 3.88)	1.57 (0.79 to 3.14)	1.73 (1.00 to 2.98)	1.51 (0.91 to 2.50)
Oral estrogen plus LNG IUS	0.83 (0.12 to 5.90)	0.85 (0.21 to 3.39)	1.14 (0.43 to 3.03)	1.15 (0.52 to 2.56)	1.59 (0.88 to 2.87)
Tibolone	2.42 (1.01 to 5.82)	1.97 (0.98 to 3.94)	2.15 (1.25 to 3.71)	1.23 (1.44 to 3.46)	2.27 (1.56 to 3.31)
Transdermal combined	0.80 (0.26 to 2.48)	0.54 (0.20 to 1.44)	0.82 (0.43 to 1.58)	1.04 (0.65 to 1.68)	1.02 (0.67 to 1.55)
Transdermal unopposed estrogen	2.55 (1.06 to 6.14)	1.86 (0.89 to 3.90)	1.98 (1.10 to 3.58)	1.71 (1.01 to 2.89)	1.66 (1.05 to 2.64)
**Cerebral infarction**					
Non-initiators	0.67 (0.65 to 0.69)	0.62 (0.60 to 0.63)	0.60 (0.59 to 0.61)	0.60 (0.59 to 0.61)	0.58 (0.57 to 0.59)
Combined continuous	1.16 (0.66 to 2.05)	0.87 (0.55 to 1.38)	0.80 (0.54 to 1.19)	0.58 (0.39 to 0.84)	0.54 (0.39 to 0.76)
Combined sequential	0.20 (0.03 to 1.39)	0.59 (0.26 to 1.30)	0.52 (0.26 to 1.04)	0.39 (0.20 to 0.75)	0.39 (0.22 to 0.68)

Oral estrogen	0.60 (0.08 to 4.26)	0.30 (0.04 to 2.12)	0.20 (0.03 to 1.41)	0.40 (0.13 to 1.23)	0.49 (0.21 to 0.19)
Oral estrogen plus LNG IUS	0.77 (0.11 to 5.46)	0.38 (0.05 to 2.72)	0.51 (0.13 to 2.03)	0.68 (0.25 to 1.81)	0.63 (0.26 to 1.52)
Tibolone	0.47 (0.07 to 3.32)	1.18 (0.49 to 2.84)	1.08 (0.51 to 2.26)	0.92 (0.48 to 1.77)	0.99 (0.58 to 1.71)
Transdermal combined	0.25 (0.03 to 1.76)	0.50 (0.19 to 1.33)	0.32 (0.12 to 0.87)	0.49 (0.25 to 0.94)	0.49 (0.28 to 0.86)
Transdermal unopposed estrogen	No events	No events	0.16 (0.02 to 1.16)	0.33 (0.10 to 1.01)	0.32 (0.12 to 0.86)
**Myocardial infarction**					
Non-initiators	0.74 (0.70 to 0.77)	0.68 (0.65 to 0.70)	0.67 (0.65 to 0.68)	0.67 (0.65 to 0.69)	0.65 (0.64 to 0.66)
Combined continuous	1.04 (0.58 to 1.89)	0.92 (0.59 to 1.45)	0.85 (.58 to 1.26)	0.69 (0.49 to 0.99)	0.70 (0.51 to 0.95)
Combined sequential	1.14 (0.51 to 2.55)	0.79 (0.39 to 1.58)	0.67 (0.36 to 1.25)	0.64 (0.38 to 1.08)	0.63 (0.40 to 1.00)
Oral estrogen	1.16 (0.29 to 4.62)	0.59 (0.15 to 2.38)	0.40 (0.10 to 1.61)	0.27 (0.07 to 1.09)	0.31 (0.10 to 0.96)
Oral estrogen plus LNG IUS	No events	No events	No events	No events	0.28 (0.07 to 1.11)
Tibolone	0.49 (0.07 to 3.46)	0.49 (0.24 to 1.99)	0.84 (0.35 to 2.02)	1.02 (0.53 to 1.97)	0.86 (0.46 to 1.60)
Transdermal combined	No events	No events	0.35 (0.13 to 0.94)	0.36 (0.16 to 0.80)	0.32 (0.25 to 0.67)
Transdermal unopposed estrogen	0.98 (0.24 to 3.90)	0.51 (0.13 to 2.04)	0.52 (0.17 to 1.62)	0.36 (0.15 to 1.11)	0.27 (0.09 to 0.84)
**Venous thromboembolism**					
Non-initiators	1.58 (1.53 to 1.63)	1.49 (1.44 to 1.54)	1.45 (1.42 to 1.48)	1.43 (1.40 to 1.45)	1.40 (1.38 to 1.44)
Combined continuous	3.70 (2.65 to 5.16)	2.83 (2.17 to 3.70)	2.70 (2.16 to 3.37)	2.29 (1.87 to 2.79)	2.16 (1.82 to 2.57)
Combined sequential	3.37 (2.09 to 5.42)	3.35 (2.39 to 4.69)	2.81 (2.08 to 3.79)	2.93 (2.31 to 3.73)	2.74 (2.22 to 3.39)
Oral estrogen	4.02 (1.80 to 8.94)	3.32 (1.79 to 6.18)	2.85 (1.66 to 4.91)	2.45 (1.52 to 3.95)	2.26 (1.47 to 3.46)
Oral estrogen plus LNG IUS	3.63 (1.51 to 8.74)	2.53 (1.21 to 5.31)	1.68 (0.80 to 3.52)	2.06 (1.20 to 3.55)	1.89 (1.16 to 3.09)
Tibolone	0.49 (0.07 to 3.51)	0.98 (0.37 to 2.61)	1.13 (0.54 to 2.37)	0.95 (0.50 to 1.83)	1.18 (0.71 to 1.96)
Transdermal combined	3.48 (2.06 to 5.89)	3.20 (2.18 to 4.71)	2.61 (1.84 to 3.69)	2.26 (1.67 to 3.05)	1.92 (1.45 to 2.55)
Transdermal unopposed estrogen	2.45 (1.02 to 5.89)	2.43 (1.31 to 4.52)	1.76 (0.98 to 3.19)	1.70 (1.04 to 2.76)	1.50 (0.95 to 2.34)

*Adjusted incidence rates include adjustment for age, trial month, calendar year, level of education, residence of living, region of birth, and medication for hypertension, heart disease and diabetes. LNG IUS = Levonorgestrel intrauterine system

Table S7. Intention-to-treat analyses with unadjusted hazard ratios, adjusted incidence rates and adjusted hazard ratios for cardiovascular disease and cause-specific cardiovascular disease with the use of menopausal hormone therapy

Type of menopausal hormone therapy	Person-years	No of events	aIR (No of events/1000 person-years)*	Unadjusted HR (95% CI)	P-value	IPW aHR (95% CI)**	P-value
**Cardiovascular disease**							
Non-initiators	100986776	239316	2.17	1 (Reference)		1 (Reference)	
Combined continuous	56898	142	2.42	1.10 (0.93 to 1.30)	0.29	1.13 (0.95 to 1.33)	0.16
Combined sequential	31353	58	2.00	0.80 (0.61 to 1.04)	0.10	0.93 (0.71 to 1.21)	0.57
Oral estrogen	8904	20	2.03	0.95 (0.61 to 1.48)	0.81	0.94 (0.60 to 1.47)	0.79
Oral estrogen plus LNG IUS	8575	16	2.26	0.85 (0.52 to 1.41)	0.53	1.06 (0.64 to 1.75)	0.81
Tibolone	12046	40	3.33	1.47 (1.07 to 2.01)	0.02	1.52 (1.11 to 2.08)	0.009
Transdermal combined	24354	34	1.54	0.62 (0.44 to 0.88)	0.006	0.69 (0.49 to 0.97)	0.04
Transdermal unopposed estrogen	12303	22	1.99	0.80 (0.52 to 1.22)	0.30	0.89 (0.58 to 1.37)	0.60
**Ischemic heart disease**							
Non-initiators	100986776	171310	1.66	1 (Reference)		1 (Reference)	
Combined continuous	56898	108	1.86	1.12 (0.93 to 1.35)	0.24	1.21 (1.00 to 1.46)	0.05
Combined sequential	31353	46	1.59	0.87 (0.65 to 1.15)	0.33	1.07 (0.80 to 1.44)	0.65
Oral estrogen	8904	15	1.51	1.00 (0.60 to 1.65)	0.98	1.00 (0.59 to 1.66)	0.98
Oral estrogen plus LNG IUS	8575	11	1.59	0.76 (0.42 to 1.37)	0.35	1.04 (0.57 to 1.91)	0.88
Tibolone	12046	27	2.27	1.32 (0.91 to 1.93)	0.15	1.46 (1.00 to 2.14)	0.05
Transdermal combined	24354	22	1.02	0.53 (0.35 to 0.81)	0.003	0.62 (0.41 to 0.96)	0.03
Transdermal unopposed estrogen	12303	18	1.66	0.86 (0.54 to 1.37)	0.53	0.98 (0.62 to 1.61)	0.99
**Cerebral infarction**							
Non-initiators	100986776	68006	0.58	1 (Reference)		1 (Reference)	
Combined continuous	56898	34	0.54	0.89 (0.63 to 1.24)	0.49	0.92 (0.66 to 1.30)	0.64

Combined sequential	31353	12	0.39	0.57 (0.32 to 1.00)	0.05	0.57 (0.31 to 1.02)	0.06
Oral estrogen	8904	5	0.49	0.83 (0.35 to 2.00)	0.69	0.81 (0.33 to 1.98)	0.74
Oral estrogen plus LNG IUS	8575	5	0.63	0.87 (0.36 to 2.08)	0.75	1.10 (0.45 to 2.69)	0.83
Tibolone	12046	13	0.99	1.60 (0.93 to 2.76)	0.09	1.66 (0.95 to 2.89)	0.07
Transdermal combined	24354	12	0.49	0.73 (0.42 to 1.29)	0.28	0.84 (0.47 to 1.52)	0.58
Transdermal unopposed estrogen	12303	4	0.32	0.48 (0.18 to 1.29)	0.15	0.61 (0.23 to 1.65)	0.33
**Myocardial infarction**							
Non-initiators	100986776	72065	0.65	1 (Reference)		1 (Reference)	
Combined continuous	56898	41	0.70	1.01 (0.74 to 1.37)	0.95	1.07 (0.79 to 1.46)	0.70
Combined sequential	31353	20	0.63	0.89 (0.58 to 1.39)	0.62	1.08 (0.69 to 1.68)	0.80
Oral estrogen	8904	3	0.31	0.47 (0.15 to 1.46)	0.19	0.44 (0.14 to 1.40)	0.18
Oral estrogen plus LNG IUS	8575	2	0.28	0.33 (0.08 to 1.31)	0.11	0.41 (0.10 to 1.78)	0.20
Tibolone	12046	10	0.86	1.16 (0.63 to 2.16)	0.63	1.25 (0.67 to 2.34)	0.62
Transdermal combined	24354	7	0.32	0.40 (0.19 to 0.85)	0.02	0.44 (0.21 to 0.94)	0.03
Transdermal unopposed estrogen	12303	3	0.27	0.34 (0.11 to 1.06)	0.06	0.59 (0.12 to 1.22)	0.10
**Venous thromboembolism**							
Non-initiators	100986776	145972	1.40	1 (Reference)		1 (Reference)	
Combined continuous	56898	128	2.16	1.56 (1.31 to 1.85)	<0.001	1.61 (1.35 to 1.92)	<0.001
Combined sequential	31353	85	2.74	1.88 (1.52 to 2.32)	<0.001	2.00 (1.61 to 2.49)	<0.001
Oral estrogen	8904	21	2.26	1.63 (1.06 to 2.50)	0.02	1.57 (1.02 to 2.44)	0.04
Oral estrogen plus LNG IUS	8575	16	1.89	1.29 (0.80 to 2.11)	0.31	1.42 (0.85 to 2.37)	0.18
Tibolone	12046	15	1.18	0.86 (0.52 to 1.43)	0.56	0.79 (0.47 to 1.34)	0.39
Transdermal combined	24354	48	1.92	1.36 (1.03 to 1.81)	0.03	1.46 (1.09 to 1.95)	0.01
Transdermal unopposed estrogen	12303	19	1.50	1.07 (0.68 to 1.68)	0.77	1.16 (0.73 to 1.83)	0.54

aIR = adjusted incidence rates. aHR = adjusted hazard ration. IPW = inverse probability weights. CI = Confidence interval. LNG IUS = levonorgestrel intrauterine system. *Adjusted incidence rates include adjustment for age, trial month, calendar year, level of education, residence of living, region of birth, and medication for hypertension, heart disease and diabetes. **Adjusted hazard ratios include inverse probability for

treatment weighting, including the same baseline covariates as the adjusted incidence rates. The hazard ratios were pooled across all trials. Since many women participated in more than one of the trials, we used the robust variance estimator to estimate the conservative 95% confidence intervals.

Table S8. Intention-to-treat analyses with adjusted subdistribution hazard ratios for cardiovascular disease and cause-specific hazard ratios with the use of menopausal hormone therapy.

Type of menopausal hormone therapy	IPW adjusted HR (95% CI)*	P-value
**Cardiovascular disease**		
Non-initiators	1 (Reference)	
Combined continuous	1.12 (0.95 to 1.32)	0.18
Combined sequential	0.94 (0.73 to 1.22)	0.65
Oral estrogen	0.98 (0.63 to 1.51)	0.92
Oral estrogen plus LNG IUS	1.03 (0.63 to 1.67)	0.92
Tibolone	1.51 (1.11 to 2.05)	0.01
Transdermal combined	0.69 (0.49 to 0.96)	0.03
Transdermal unopposed estrogen	0.88 (0.58 to 1.34)	0.56
**Ischemic heart disease**		
Non-initiators	1 (Reference)	
Combined continuous	1.19 (0.99 to 1.44)	0.07
Combined sequential	1.05 (0.89 to 1.40)	0.75
Oral estrogen	1.02 (0.62 to 1.70)	0.93
Oral estrogen plus LNG IUS	0.99 (0.55 to 1.78)	0.96
Tibolone	1.42 (0.97 to 2.07)	0.06
Transdermal combined	0.62 (0.41 to 0.95)	0.03
Transdermal unopposed estrogen	1.01 (0.64 to 1.61)	0.96
**Cerebral infarction**		
Non-initiators	1 (Reference)	

Combined continuous	0.94 (0.67 to 1.32)	0.72
Combined sequential	0.68 (0.39 to 1.20)	0.18
Oral estrogen	0.86 (0.36 to 2.06)	0.73
Oral estrogen plus LNG IUS	1.12 (0.47 to 2.69)	0.80
Tibolone	1.72 (1.00 to 2.97)	0.05
Transdermal combined	0.85 (0.48 to 1.50)	0.58
Transdermal unopposed estrogen	0.56 (0.21 to 1.50)	0.25
**Myocardial infarction**		
Non to initiators	1 (Reference)	
Combined continuous	1.08 (0.79 to 1.46)	0.63
Combined sequential	1.09 (0.70 to 1.69)	0.70
Oral estrogen	0.49 (0.16 to 1.51)	0.21
Oral estrogen plus LNG IUS	0.44 (0.11 to 1.74)	0.24
Tibolone	1.27 (0.68 to 2.36)	0.45
Transdermal combined	0.48 (0.23 to 1.00)	0.06
Transdermal unopposed estrogen	0.41 (0.13 to 1.27)	0.12
**Venous thromboembolism**		
Non to initiators	1 (Reference)	
Combined continuous	1.59 (1.34 to 1.89)	<0.001
Combined sequential	2.03 (1.64 to 2.51)	<0.001
Oral estrogen	1.65 (1.08 to 2.54)	0.02
Oral estrogen plus LNG IUS	1.44 (0.88 to 2.34)	0.15
Tibolone	0.88 (0.53 to 1.47)	0.64
Transdermal combined	1.45 (1.09 to 1.93)	0.01
Transdermal unopposed estrogen	1.14 (0.73 to 1.78)	0.57

IPW = inverse probability weights. CI = Confidence interval. LNG IUS = levonorgestrel intrauterine system. *Adjusted hazard ratios include inverse probability for treatment weighting, including the baseline covariates age, trial month, calendar year, level of education, residence of

living, region of birth, and medication for hypertension, heart disease and diabetes. The hazard ratios were pooled across all trials. Since many women participated in more than one of the trials, we used the robust variance estimator to estimate the conservative 95% confidence intervals.

Table S9. Intention-to-treat analyses with adjusted hazard ratios for cardiovascular disease and cause-specific hazard ratios with use of menopausal hormone therapy with study period 2011 to 2020.

Type of menopausal hormone therapy	Person-years	No of events	IPW adjusted HR (95% CI)*	P-value
**Cardiovascular disease**				
Non-initiators	63018864	137645	1 (Reference)	
Combined continuous	32155	71	1.09 (0.87 to 1.38)	0.45
Combined sequential	17710	30	0.95 (0.67 to 1.36)	0.79
Oral estrogen	3433	8	1.14 (0.57 to 2.27)	0.72
Oral estrogen plus LNG IUS	3080	5	0.98 (0.41 to 2.36)	0.96
Tibolone	5792	11	1.00 (0.55 to 1.80)	0.99
Transdermal combined	13212	14	0.59 (0.35 to 0.99)	0.05
Transdermal unopposed estrogen	5984	4	0.37 (0.14 to 0.99)	0.05
**Ischemic heart disease**				
Non-initiators	63018864	96317	1 (Reference)	
Combined continuous	32155	55	1.22 (0.93 to 1.58)	0.15
Combined sequential	17710	21	0.97 (0.63 to 1.48)	0.87
Oral estrogen	3433	7	1.42 (0.68 to 2.98)	0.35
Oral estrogen plus LNG IUS	3080	4	1.14 (0.43 to 3.03)	0.79
Tibolone	5792	6	0.79 (0.35 to 1.75)	0.55
Transdermal combined	13212	8	0.49 (0.24 to 0.97)	0.04
Transdermal unopposed estrogen	5984	4	0.54 (0.20 to 1.43)	0.21
**Cerebral infarction**				
Non-initiators	63018864	41328	1 (Reference)	
Combined continuous	32155	16	0.81 (0.50 to 1.33)	0.41

Combined sequential	17710	9	0.93 (0.48 to 1.79)	0.82
Oral estrogen	3433	1	0.47 (0.07 to 3.35)	0.45
Oral estrogen plus LNG IUS	3080	1	0.63 (0.09 to 4.45)	0.65
Tibolone	5792	5	1.47 (0.61 to 3.54)	0.39
Transdermal combined	13212	6	0.82 (0.37 to 1.82)	0.62
Transdermal unopposed estrogen	5984	0		
**Myocardial infarction**				
Non-initiators	63018864	43540	1 (Reference)	
Combined continuous	32155	22	1.08 (0.71 to 1.64)	0.71
Combined sequential	17710	9	0.93 (0.48 to 1.78)	0.82
Oral estrogen	3433	2	0.90 (0.22 to 3.60)	0.88
Oral estrogen plus LNG IUS	3080	0		
Tibolone	5792	0		
Transdermal combined	13212	3	0.41 (0.13 to 1.29)	0.13
Transdermal unopposed estrogen	5984	0		
**Venous thromboembolism**				
Non-initiators	63018864	90322	1 (Reference)	
Combined continuous	32155	78	1.74 (1.39 to 2.17)	<0.001
Combined sequential	17710	43	1.83 (1.36 to 2.47)	<0.001
Oral estrogen	3433	10	2.09 (1.12 to 3.89)	0.02
Oral estrogen plus LNG IUS	3080	5	1.26 (0.52 to 3.02)	0.61
Tibolone	5792	7	0.87 (0.42 to 1.82)	0.72
Transdermal combined	13212	26	1.46 (1.00 to 2.15)	0.05
Transdermal unopposed estrogen	5984	7	0.87 (0.41 to 1.82)	0.71

IPW = inverse probability weights. CI = Confidence interval. LNG IUS = levonorgestrel intrauterine system. *Adjusted hazard ratios include inverse probability for treatment weighting, including the baseline covariates age, trial month, calendar year, level of education, residence of

living, region of birth, and medication for hypertension, heart disease and diabetes. The hazard ratios were pooled across all trials. Since many women participated in more than one of the trials, we used the robust variance estimator to estimate the conservative 95% confidence intervals.

Table S10. Per-protocol analyses with unadjusted hazard ratios, adjusted incidence rates and adjusted hazard ratios for cardiovascular disease and cause-specific cardiovascular disease with the use of menopausal hormone therapy.

Type of hormonal contraception	Person-years	No of events	aIR (No of events / 1000 person-years)	Unadjusted HR (95% CI)	P-value	IPW aHR (95% CI)**	P-value
**Cardiovascular disease**							
Never user	99423179	236033	2.17	1 (Reference)		1 (Reference)	
Combined continuous	37262	101	2.67	1.14 (0.93 to 1.40)	0.22	1.22 (1.00 to 1.50)	0.05
Combined sequential	18661	34	1.98	0.75 (0.53 to 1.06)	0.11	0.91 (0.64 to 1.29)	0.61
Oral estrogen	4603	14	2.55	1.18 (0.70 to 2.00)	0.53	1.14 (0.66 to 1.96)	0.65
Oral estrogen plus LNG IUS	6076	12	2.84	0.97 (0.53 to 1.76)	0.92	1.34 (0.73 to 2.43)	0.34
Tibolone	7654	30	3.98	1.64 (1.13 to 2.36)	0.008	1.81 (1.25 to 2.61)	0.001
Transdermal combined	13213	18	1.48	0.56 (0.35 to 0.90)	0.02	0.66 (0.41 to 1.06)	0.08
Transdermal unopposed estrogen	6987	12	1.79	0.67 (0.38 to 1.18)	0.17	0.82 (0.47 to 1.45)	0.50
**Ischemic heart disease**							
Never user	99423179	168928	1.48	1 (Reference)		1 (Reference)	
Combined continuous	37262	76	1.92	1.21 (0.97 to 1.51)	0.09	1.27 (1.01 to 1.60)	0.04
Combined sequential	18661	29	1.62	0.92 (0.64 to 1.33)	0.67	1.09 (0.76 to 1.58)	0.65
Oral estrogen	4603	11	2.01	1.42 (0.79 to 2.57)	0.24	1.29 (0.70 to 2.40)	0.42
Oral estrogen plus LNG IUS	6076	8	1.57	0.78 (0.39 to 1.56)	0.48	1.05 (0.53 to 2.12)	0.87
Tibolone	7654	21	2.67	1.63 (1.06 to 2.50)	0.03	1.76 (1.14 to 2.70)	0.009
Transdermal combined	13213	13	1.05	0.59 (0.34 to 1.01)	0.05	0.67 (0.39 to 1.16)	0.15
Transdermal unopposed estrogen	6987	9	1.40	0.77 (0.40 to 1.47)	0.42	0.82 (0.48 to 1.78)	0.81
**Cerebral infarction**							

Never user	99423179	67105	0.57	1 (Reference)		1 (Reference)	
Combined continuous	37262	25	0.61	1.01 (0.68 to 1.49)	0.96	1.08 (0.73 to 1.60)	0.70
Combined sequential	18661	5	0.27	0.41 (0.17 to 0.98)	0.04	0.49 (0.20 to 1.19)	0.11
Oral estrogen	4603	3	0.55	1.00 (0.32 to 3.07)	0.99	1.02 (0.33 to 3.15)	0.98
Oral estrogen plus LNG IUS	6076	4	0.72	0.99 (0.37 to 2.63)	0.98	1.36 (0.51 to 3.63)	0.54
Tibolone	7654	9	1.09	1.77 (0.92 to 3.41)	0.09	1.97 (1.02 to 3.78)	0.04
Transdermal combined	13213	5	0.37	0.58 (0.24 to 1.38)	0.22	0.64 (0.27 to 1.55)	0.32
Transdermal unopposed estrogen	6987	3	0.43	0.65 (0.21 to 2.01)	0.46	0.78 (0.25 to 2.42)	0.67
**Myocardial infarction**							
Never user	99423179	71159	0.62	1 (Reference)		1 (Reference)	
Combined continuous	37262	34	0.84	1.29 (0.92 to 1.81)	0.13	1.38 (0.99 to 1.94)	0.06
Combined sequential	18661	13	0.73	1.00 (0.58 to 1.71)	0.98	1.22 (0.71 to 2.11)	0.47
Oral estrogen	4603	2	0.37	0.62 (0.16 to 2.48)	0.5	0.64 (0.16 to 2.55)	0.52
Oral estrogen plus LNG IUS	6076	2	0.38	0.45 (0.12 to 1.86)	0.28	0.63 (0.15 to 2.54)	0.52
Tibolone	7654	9	1.16	1.67 (0.87 to 3.21)	0.12	1.94 (1.01 to 3.73)	0.04
Transdermal combined	13213	4	0.32	0.43 (0.16 to 1.15)	0.09	0.50 (0.19 to 1.35)	0.18
Transdermal unopposed estrogen	6987	1	0.15	0.20 (0.03 to 1.45)	0.11	0.25 (0.04 to 1.77)	0.17
**Venous thromboembolism**							
Never user	99423179	142445	1.28	1 (Reference)		1 (Reference)	
Combined continuous	37262	96	2.34	1.84 (1.50 to 2.24)	<0.001	1.84 (1.50 to 2.25)	<0.001
Combined sequential	18661	58	2.99	2.23 (1.73 to 2.89)	<0.001	2.45 (1.89 to 3.17)	<0.001
Oral estrogen	4603	10	1.94	1.57 (0.84 to 2.91)	0.15	1.59 (0.86 to 2.97)	0.14
Oral estrogen plus LNG IUS	6076	12	1.91	1.40 (0.79 to 2.46)	0.25	1.60 (0.91 to 2.83)	0.10
Tibolone	7654	8	0.94	0.78 (0.37 to 1.49)	0.41	0.76 (0.38 to 1.53)	0.45
Transdermal combined	13213	29	2.01	1.58 (1.10 to 2.28)	0.01	1.67 (1.16 to 2.41)	0.006
Transdermal unopposed estrogen	6987	10	1.31	1.03 (0.55 to 1.91)	0.93	1.09 (0.59 to 2.03)	0.78

aIR = adjusted incidence rates. aHR = adjusted hazard ratio. IPW = inverse probability weights. CI = Confidence interval. LNG IUS = levonorgestrel intrauterine system. *Adjusted incidence rates include adjustment for age, trial month, calendar year, level of education, residence of living, region of birth, and medication for hypertension, heart disease and diabetes. **Adjusted hazard ratios include inverse probability for treatment and censoring weighting, including the same baseline covariates as the adjusted incidence rates and medication status as a time-varying covariate. The hazard ratios were pooled across all trials. Since many women participated in more than one of the trials, we used the robust variance estimator to estimate the conservative 95% confidence intervals.
